# Advances in quantitative ultrasound for metabolic dysfunction-associated steatotic liver disease diagnosis

**DOI:** 10.3389/fphys.2026.1802284

**Published:** 2026-05-08

**Authors:** Zejun Ma, Bo Liu, Shuyu Zhou, Shuai Yang, Xiaojie Sun, Xinping Jiang, Xiaofeng Sun

**Affiliations:** 1 Department of Cadre's Wards Ultrasound Diagnostics, Ultrasound Diagnostic Center, The First Hospital of Jilin University, Changchun, China; 2 The First Hospital of Jilin University, Changchun, China

**Keywords:** artificial intelligence, attenuation imaging, liver physiology, MASLD, quantitative ultrasound, shear-wave elastography

## Abstract

Metabolic dysfunction–associated steatotic liver disease (MASLD) is increasing worldwide, underscoring the need for noninvasive, repeatable biomarkers for accurate clinical stratification. This review synthesizes recent advances in quantitative ultrasound (QUS) for MASLD phenotyping, spanning attenuation-based metrics (e.g., controlled attenuation parameter, tissue attenuation imaging, ultrasound-guided attenuation parameter), shear-wave elastography, backscatter analysis, sound-speed imaging, and artificial-intelligence–enabled multiparametric models. Unlike conventional B-mode ultrasound, which relies on subjective echogenicity, QUS derives biophysical parameters from radiofrequency signals that correlate with hepatic fat content, stiffness and viscoelasticity, microstructure, and perfusion. Across recent evidence, attenuation techniques show strong performance for detecting early steatosis, whereas shear-wave elastography better stages clinically significant fibrosis; shear-wave dispersion and microvascular imaging provide emerging surrogates of necroinflammatory activity. Integrating multiple QUS features with clinical covariates improves robustness and reduces diagnostic error relative to single-parameter tools. We summarize technical principles, acquisition considerations, and sources of variability, and discuss harmonization, cross-platform comparability, and the role of open protocols. Remaining challenges include vendor heterogeneity, the absence of unified thresholds, limited multicenter outcome data, and the need for explainable and generalizable AI. Overall, QUS is evolving from a screening adjunct to a physiologically grounded, multiparametric platform. However, current challenges such as cross-platform harmonization, vendor variability, and the need for unified diagnostic thresholds remain significant barriers. The future of QUS lies in the integration of artificial intelligence (AI) to enhance diagnostic accuracy, improve reproducibility, and address these limitations. Further research should focus on large-scale validation studies and the development of multi-parametric approaches that combine QUS with other non-invasive diagnostic tools for a more comprehensive assessment of MASLD.

## Highlights

MASLD is becoming increasingly prevalent, driven by rising obesity and metabolic syndrome rates worldwide. Early and accurate diagnosis of MASLD, particularly in its early stages of steatosis, is crucial for effective disease management and prevention of progression to advanced stages like cirrhosis or hepatocellular carcinoma. This review highlights the role of quantitative ultrasound (QUS) in improving the non-invasive assessment of MASLD, with a focus on advanced techniques like attenuation imaging, shear-wave elastography, and artificial intelligence (AI)-enabled models. Our findings suggest that QUS methods offer significant diagnostic advantages over conventional ultrasound, providing objective, reproducible biomarkers that correlate with liver fat content, tissue stiffness, and microvascular changes. The integration of multiple QUS modalities with clinical covariates has the potential to enhance diagnostic accuracy and stratify patients more effectively, reducing diagnostic error and guiding therapeutic decisions. This work underscores the potential of QUS as a tool for personalized management of MASLD, with implications for improving early detection, monitoring disease progression, and tailoring treatment plans.

## Introduction

Metabolic dysfunction-associated steatotic liver disease (MASLD), formerly known as non-alcoholic fatty liver disease (NAFLD), has rapidly become the most prevalent chronic liver condition worldwide, paralleling the global rise of obesity, insulin resistance, and metabolic syndrome (Pipitone et al., 2023). MASLD is a more comprehensive term, encompassing a spectrum of liver diseases that are linked to metabolic dysfunction, such as insulin resistance and obesity, while NAFLD primarily refers to fatty liver in the absence of alcohol consumption. Despite both terms addressing liver steatosis, MASLD emphasizes the underlying metabolic causes and includes stages of disease progression from simple steatosis to steatohepatitis (MASH), fibrosis, cirrhosis, and hepatocellular carcinoma (Byrne and Targher, 2015).

While fibrosis is the primary determinant of prognosis in MASLD, steatosis grading still holds significant clinical relevance. Recent studies suggest that grading hepatic steatosis is crucial for the early diagnosis and monitoring of liver disease progression, especially in the early stages of disease. The degree of fat deposition in the liver is directly linked to alterations in liver metabolism and subsequent pathological changes, such as apoptosis and inflammatory responses, which are detectable by imaging techniques at early stages of the disease.

Although fibrosis has a more direct impact on prognosis, early identification of steatosis allows for better precision stratification of patients, enabling timely intervention and preventing further progression to cirrhosis or hepatocellular carcinoma (HCC). Therefore, steatosis grading plays an essential role not only in understanding the current status of the liver but also in informing future treatment decisions. Particularly, in the absence of liver biopsy, non-invasive imaging tools such as QUS (Quantitative Ultrasound) can effectively assess hepatic fat and provide valuable insights for disease management.

Accurate characterization of hepatic steatosis, inflammation, and fibrosis is essential for early diagnosis, prognostication, and treatment monitoring in MASLD (Abdlaty et al., 2025). Liver biopsy remains the reference standard for histological staging but is limited by invasiveness, sampling variability, and poor acceptance in asymptomatic populations. Magnetic resonance-based techniques such as proton density fat fraction (MRI-PDFF) and elastography (MRE) provide non-invasive quantitative assessment but are expensive and not widely accessible. Conventional B-mode ultrasound is widely used for liver screening, yet its diagnostic accuracy is limited by operator dependence and subjective interpretation (Eslam et al., 2020b).

Quantitative ultrasound (QUS) technologies have emerged as promising tools for the noninvasive characterization of liver tissue by extracting measurable acoustic biomarkers related to hepatic fat content, stiffness, and microstructure. These techniques can be broadly categorized according to their dominant physical measurement principles. Attenuation-based approaches, such as the controlled attenuation parameter (CAP), tissue attenuation imaging (TAI), and ultrasound-guided attenuation parameter (UGAP), primarily quantify ultrasound energy loss associated with lipid accumulation. Elasticity-based techniques, including shear wave elastography (SWE), evaluate tissue stiffness and are widely used for fibrosis assessment. Backscatter-based methods analyze echo signal properties related to tissue microstructure, while sound-speed imaging estimates acoustic propagation velocity associated with tissue composition.

Although several quantitative ultrasound approaches rely on analysis of radiofrequency (RF) signals, many clinically implemented systems derive parameters from envelope-detected signals or other processed data depending on platform design. These complementary modalities therefore reflect different acoustic properties of liver tissue and can be linked to specific physiological or pathological features of MASLD.

QUS offers practical advantages including non-invasiveness, portability, real-time imaging, and cost-efficiency, making it suitable for primary care and longitudinal surveillance (Eslam et al., 2020a; Méndez-Sánchez et al., 2022).

**Figure 1:**
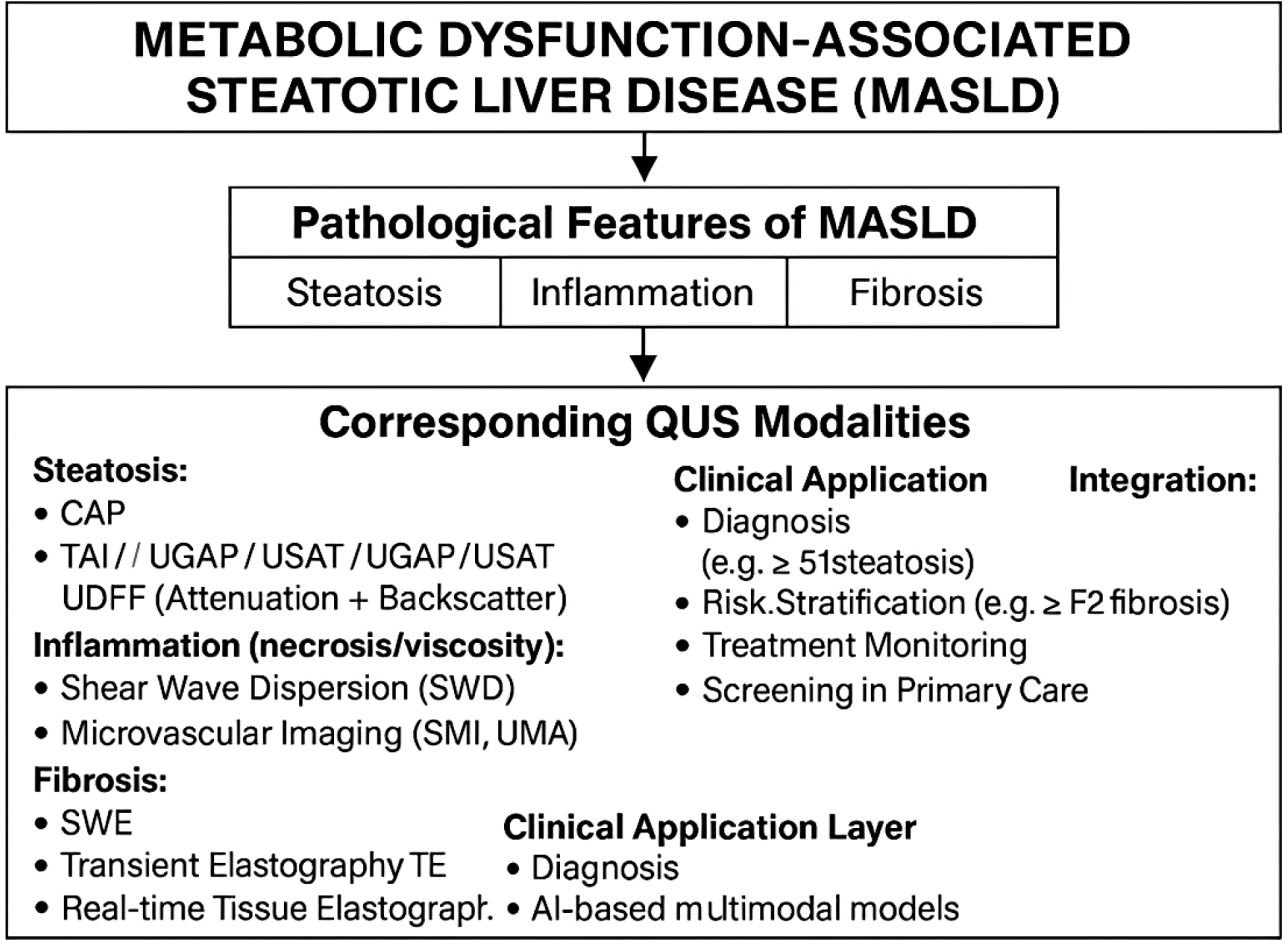
Schematic illustration of QUS modalities applied to MASLD diagnosis, mapping techniques (e.g., CAP, TAI, SWE, AI) to corresponding pathological targets: steatosis, fibrosis, and inflammation.

Despite increasing clinical adoption, several challenges limit the broader implementation of QUS. First, the lack of standardized algorithms across vendors introduces variability in results, which complicates multi-center studies and clinical practice. To overcome this, future research should focus on the development of universal calibration protocols and the establishment of standardized diagnostic thresholds. Additionally, integrating artificial intelligence (AI) to assist in feature extraction and automated analysis can improve accuracy and mitigate inter-operator variability. AI-enhanced QUS may play a pivotal role in the next generation of diagnostic tools, helping to bridge the gap between existing technological limitations and the clinical need for precision medicine in MASLD (Song et al., 2024). In addition, most studies focus on single-parameter QUS tools, while emerging multiparametric approaches—including ultrasound-derived fat fraction (UDFF) and AI-enhanced image fusion—remain underexplored. From a physiological perspective, there remains a need to align QUS biomarkers with the mechanistic understanding of MASLD progression, particularly regarding necroinflammatory activity and microvascular remodeling. Artificial intelligence (AI) offers important opportunities to integrate multiple QUS-derived parameters and enhance diagnostic robustness, but challenges such as data set limitations, generalizability, and explainability need to be addressed. As illustrated in **Figure 1**, QUS modalities for MASLD diagnosis map specific techniques to corresponding pathological targets.

This review aims to provide a comprehensive classification of QUS technologies used in MASLD diagnosis; summarize their technical foundations, diagnostic performance, and physiological relevance across disease stages; and highlight unmet needs and future directions, including multiparametric integration, cross-platform standardization, and AI-assisted phenotyping. By bridging imaging physics, liver physiology, and clinical hepatology, we propose that QUS has the potential to evolve from a screening tool into a physiological biomarker platform for precision management of MASLD.

## Conventional and quantitative ultrasound: from B-mode to Biophysics

Conventional B-mode examinations visualize macroscopic morphology and coarse echotexture, offering rapid, radiation-free screening at the point of care. In the context of MASLD, increased echogenicity, posterior beam attenuation, and vascular blurring remain the classic sonographic hallmarks of hepatic steatosis. Yet these criteria are inherently subjective and require considerable operator experience, resulting in wide inter-observer variability and limited sensitivity for early or mild disease (Bohte et al., 2011).

In addition to purely visual assessment, several semi-quantitative ultrasound scoring systems have been proposed to improve the objectivity of conventional ultrasound evaluation. Methods such as the Hamaguchi score, the Ultrasound Fatty Liver Index (US-FLI), and the hepatorenal index (HRI) attempt to quantify hepatic steatosis by integrating grayscale features, echogenicity differences between the liver and kidney, vascular blurring, and posterior beam attenuation. HRI, for example, compares the relative brightness of the liver and kidney to assess steatosis, offering a semi-quantitative approach to liver fat evaluation. Unlike RF-based backscatter techniques, which analyze tissue microstructure, HRI relies on echogenicity differences and is therefore operator-dependent. The hepatorenal index (HRI) is based on a semi-automated or automated assessment of ultrasound image brightness, allowing calculation of a quantitative liver-to-kidney echogenicity ratio; although this approach improves objectivity compared with purely visual assessment, variability may still arise from factors such as machine settings and region-of-interest placement. These approaches provide a more structured assessment compared with subjective visual interpretation and have demonstrated moderate diagnostic performance for detecting hepatic steatosis in clinical studies. Although semi-quantitative methods improve reproducibility compared with purely qualitative assessment, they remain limited by operator dependence and machine-specific settings, which has driven the development of fully quantitative ultrasound techniques.

While conventional ultrasound demonstrates moderate diagnostic accuracy for detecting moderate-to-severe hepatic steatosis, its sensitivity decreases in cases of mild fat accumulation. Several meta-analyses have reported that the diagnostic performance of conventional B-mode ultrasound becomes limited when hepatic fat infiltration is relatively low, typically in the range of approximately 20–30% hepatic fat content (Lee and Park, 2014; Xiao et al., 2017). More critically, traditional B-mode scans offer no insight into other essential physiological parameters of liver disease, including tissue stiffness, parenchymal viscosity, or microvascular remodeling—features that are increasingly recognized as central to the pathogenesis and risk stratification of MASLD. This diagnostic limitation has fueled growing interest in QUS technologies, which extract numerical data from raw acoustic signals to characterize underlying tissue properties beyond grayscale morphology (Saadeh et al., 2002).

**Figure 2:**
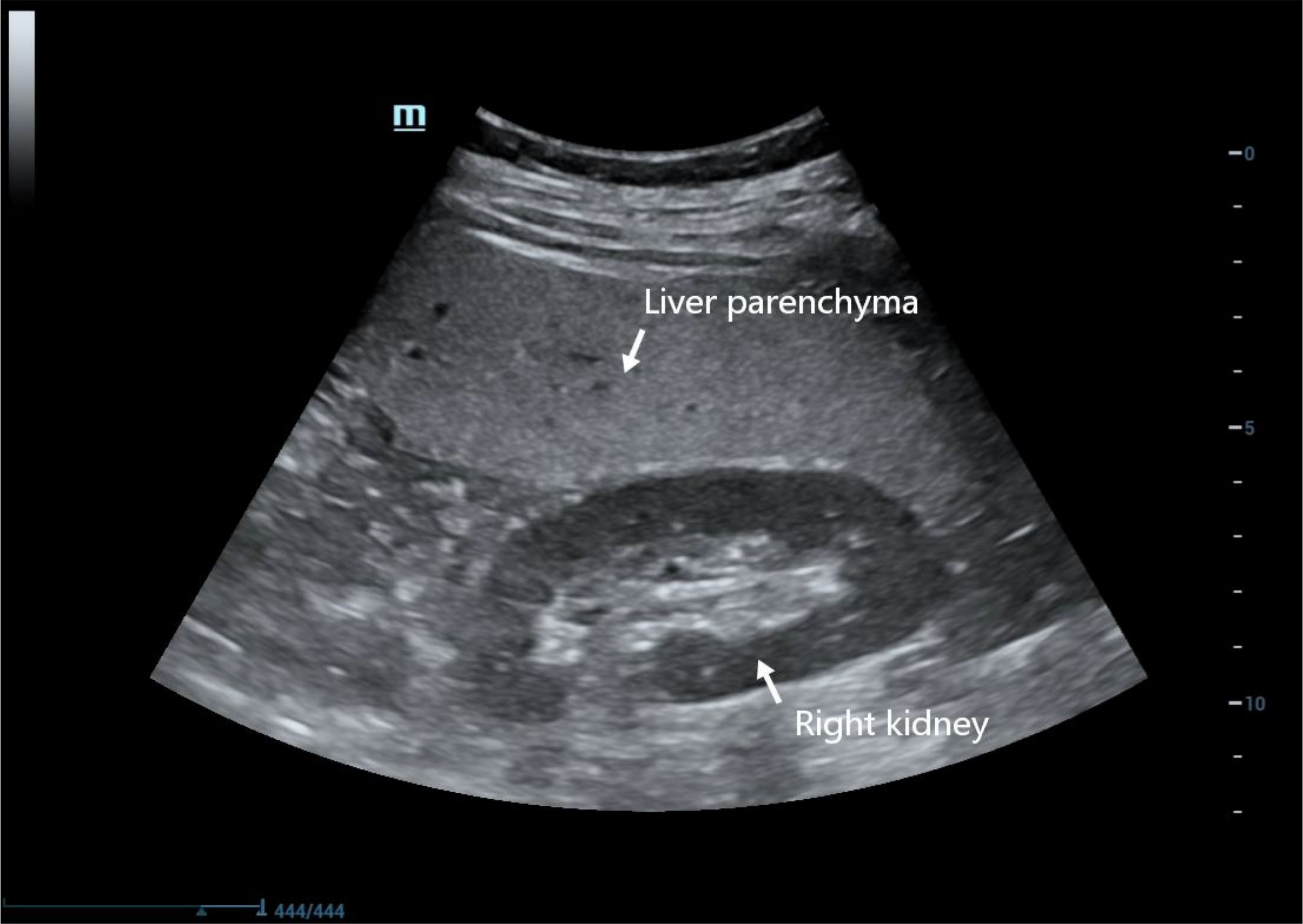
Conventional B-mode ultrasound image of the liver. The grayscale image demonstrates typical hepatic parenchyma (annotated) adjacent to the right kidney (annotated), allowing visual comparison of echogenicity between the liver and renal cortex, a commonly used reference in ultrasound assessment of hepatic steatosis. While conventional B-mode imaging provides important anatomical context, it does not offer direct quantitative assessment of hepatic fat, fibrosis, or microstructural tissue properties.

As illustrated in the revised **Figure 2**, conventional B-mode ultrasound demonstrates the typical grayscale appearance of hepatic parenchyma together with the adjacent right kidney, enabling visual comparison of echogenicity between the two structures. While this imaging approach provides useful anatomical information, it remains inherently qualitative and therefore limited in its ability to quantify liver tissue composition.

Unlike B-mode imaging, which relies on post-processed echo amplitude for visual assessment, QUS leverages radiofrequency signal data to generate quantitative biomarkers that reflect specific biophysical attributes of liver tissue. These include frequency-dependent signal attenuation influenced by lipid content, shear-wave propagation velocity associated with tissue elasticity and fibrosis, echo backscatter patterns shaped by cellular microarchitecture, and sound speed variations determined by overall tissue density and compressibility. By capturing these diverse signal components, QUS offers a physiologically grounded framework for assessing steatosis, fibrosis, inflammation, and perfusion in a manner that is objective, reproducible, and minimally operator-dependent (Ferraioli et al., 2021).

Technological advances over the past decade have led to the integration of QUS algorithms into commercial ultrasound platforms, allowing the concurrent acquisition of B-mode images and parametric maps within a single examination session. This development has expanded the clinical utility of QUS beyond research settings, enabling longitudinal monitoring and risk stratification of MASLD in routine practice. Moreover, the physiologic specificity of QUS modalities aligns with emerging demands for non-invasive tools that can not only detect hepatic fat but also monitor disease progression, treatment response, and microcirculatory alterations—all of which play critical roles in MASLD’s heterogeneous clinical course.

In the subsequent sections, we systematically review the major QUS techniques currently used in MASLD evaluation. For each category, we summarize the underlying physical principle, interpretive parameters, and clinical relevance to liver physiology and pathology. Where applicable, diagnostic performance, technical limitations, and inter-vendor variability are also discussed. This review aims to provide a comprehensive yet focused guide to clinicians and researchers navigating the expanding field of quantitative liver imaging.

**Figure 3:**
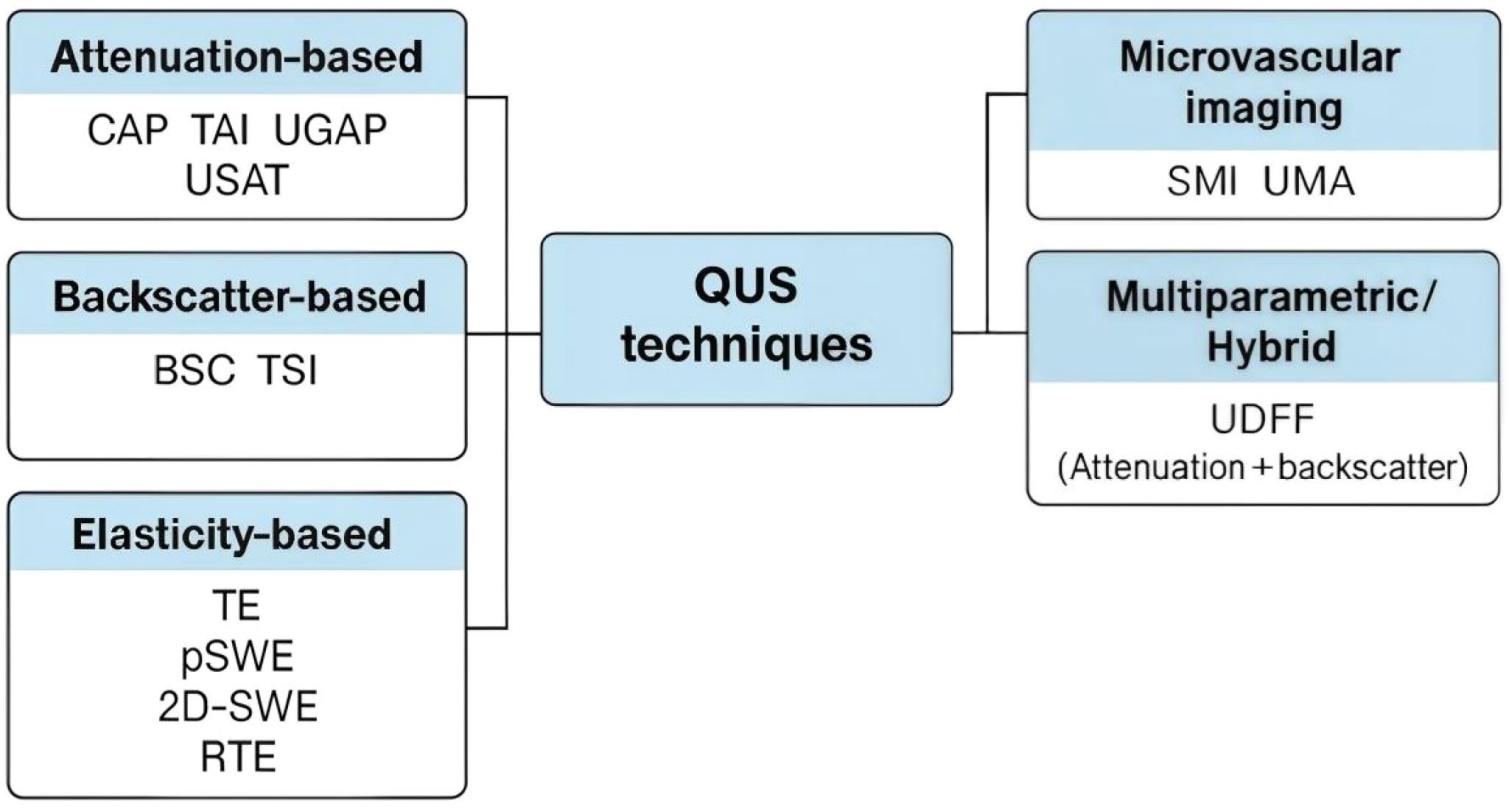
This figure illustrates the various quantitative ultrasound (QUS) techniques used for the diagnosis of Metabolic Dysfunction-Associated Steatotic Liver Disease (MASLD), categorized according to their primary measurement principles. The Attenuation-based techniques include Controlled Attenuation Parameter (CAP), Tissue Attenuation Imaging (TAI), Ultrasound-Guided Attenuation Parameter (UGAP), and Ultrasound Attenuation Analysis (USAT). The Backscatter-based techniques consist of Backscattering Coefficient (BSC), Tissue Scattering Imaging (TSI). The Elasticity-based techniques are Transient Elastography (TE), Point Shear Wave Elastography (pSWE), Two-Dimensional Shear Wave Elastography (2D-SWE), and Real-Time Tissue Elastography (RTE). Microvascular imaging includes Superb Microvascular Imaging (SMI) and Ultrasensitive Microvascular Analysis (UMA). Lastly, Multiparametric/Hybrid techniques involve Ultrasound-Derived Fat Fraction (UDFF), which combines Attenuation and Backscatter techniques, and AI-based composite models. These techniques utilize various physical principles to provide multidimensional assessments for the diagnosis of MASLD.

As illustrated in **Figure 3**, QUS modalities for MASLD can be grouped by their dominant biophysical measurement principles—such as attenuation, elasticity, backscatter, or speed of sound. However, some techniques such as UDFF or AI-assisted models combine multiple physical domains and are therefore categorized as hybrid or multiparametric approaches.

### Attenuation-based QUS for fat quantification

QUS techniques have become central to non-invasive hepatic fat quantification due to their capacity to exploit the acoustic effects of lipid accumulation. As triglycerides deposit within hepatocytes, they increase the heterogeneity of tissue acoustic impedance, leading to greater ultrasound energy dissipation. This frequency-dependent attenuation provides a physiologically meaningful biomarker of hepatic steatosis, and has thus been widely explored as a practical alternative to liver biopsy and MRI-based fat quantification.

### Controlled attenuation parameter

CAP, embedded within the FibroScan Echosens, was the first clinically validated QUS modality to quantify liver fat content (Ferraioli and Soares Monteiro, 2019). It measures ultrasound attenuation at a fixed frequency of 3.5 MHz during transient elastography, generating a single numerical value without the need for anatomical image guidance (Wang et al., 2015; Ren et al., 2021; Atzori et al., 2023).The technique has been extensively validated in diverse populations, with reported AUCs ranging from 0.82 to 0.91 for detecting ≥S1 steatosis, and demonstrates consistent agreement with both MRI-PDFF and histological grading (Das et al., 2017).

From a practical standpoint, CAP is well suited for large-scale screening and primary care use, given its operational simplicity, rapid acquisition, and vendor-standardized output. However, its blind acquisition protocol introduces vulnerability in patients with high Body Mass Index(BMI), narrow intercostal windows, or ascites, where signal penetration and localization may be compromised. Additionally, the fixed-point nature of the measurement limits its utility in detecting spatial heterogeneity or monitoring regional changes over time. Despite these drawbacks, CAP continues to serve as a foundational QUS tool, particularly where cost-effectiveness and throughput are prioritized (de Lédinghen et al., 2014).

### Tissue attenuation imaging

TAI, developed by Samsung, enhances attenuation assessment by integrating parametric mapping with real-time B-mode ultrasound (Ferraioli and Soares Monteiro, 2019; Park et al., 2022; Tamaki et al., 2022). It calculates the attenuation coefficient in decibels per centimeter per megahertz (dB/cm/MHz), allowing users to visualize and manually select the region of interest (ROI) within liver parenchyma (Pirmoazen et al., 2022). The approach retains the same biophysical foundation as CAP—measuring lipid-induced energy decay—but provides anatomical guidance and operator feedback (Lee, 2021).

Clinical studies have shown that TAI achieves diagnostic performance comparable to MRI-PDFF, with AUCs typically between 0.84 and 0.93 across various steatosis grades. The ability to avoid vascular structures and heterogeneous regions under visual control improves reproducibility, particularly in obese or anatomically complex patients (Kwon et al., 2021). However, the performance of TAI may still be influenced by rib shadowing and sonographer expertise, which can affect ROI placement and map stability. Nevertheless, its capacity for targeted fat assessment and spatial granularity positions it as a favorable option for both diagnosis and longitudinal disease monitoring (Kuroda et al., 2021; Starekova et al., 2021).

### Ultrasound-guided attenuation parameter

UGAP is an image-guided attenuation assessment technique implemented on different commercial ultrasound platforms. By enabling ROI placement under real-time B-mode guidance, it allows attenuation coefficient estimation within selected liver parenchyma while reducing interference from vessels, subcutaneous tissue, and focal heterogeneity. Like other attenuation-based methods, UGAP is based on the principle that lipid accumulation increases ultrasound energy loss during propagation. Compared with blind attenuation measurements, image-guided approaches may provide improved measurement consistency and better anatomical targeting (Bende et al., 2021; Huang et al., 2025). The technique enables users to customize ROI depth and positioning within real-time grayscale images, thereby minimizing interference from subcutaneous tissue or large vessels (Fujiwara et al., 2018; Ito, 2019).

Compared with blind techniques, UGAP offers enhanced measurement consistency, and has shown moderate correlations (r = 0.55–0.65) with MRI-PDFF and histologic steatosis scores (Moret et al., 2022; Wu et al., 2024). It is particularly advantageous in patients with regional fat heterogeneity or nonuniform liver architecture (Iwashita et al., 2022). However, the method’s diagnostic thresholds remain poorly standardized across populations, and its clinical availability is restricted to specific vendor platforms. Despite these challenges, UGAP provides greater control over signal acquisition and may be preferable in specialty settings where precision is critical.

### Ultrasound attenuation analysis

USAT, developed by Mindray, is an attenuation-based quantitative ultrasound technique that generates spatially resolved 2D liver attenuation maps (Newsome et al., 2020; Wang et al., 2024). The method estimates tissue attenuation indirectly from radiofrequency signal analysis, providing a comprehensive assessment of hepatic fat distribution while minimizing operator dependency (Newsome et al., 2020).

Initial studies have demonstrated good agreement between USAT, CAP, and MRI-PDFF, suggesting its potential role in both diagnosis and follow-up assessment (Huang et al., 2021). Its minimized reliance on operator judgment makes it especially promising in multicenter or longitudinal trials. Nevertheless, limited international validation, absence of guideline-endorsed thresholds, and restricted commercial availability currently limit its broader application. Even so, USAT provides a spatially resolved and physiologically informed approach for liver fat quantification, especially in diffuse or heterogeneous steatosis.

Attenuation-based QUS methods differ in their data acquisition strategies and algorithmic implementations. UGAP is based on raw radiofrequency (RF) data, which provides direct access to the acoustic signal before envelope detection. This allows for a more accurate estimation of tissue attenuation by reducing the influence of system-dependent post-processing. In contrast, TAI and USAT are primarily derived from envelope data rather than RF signals, although some recent vendor-specific implementations attempt to incorporate RF-based approaches. Envelope-based methods are advantageous due to easier integration into conventional scanners, but they may be more susceptible to operator and device variability.

From a technical standpoint, attenuation-based QUS techniques differ in their signal-processing strategies and levels of system integration. UGAP is typically derived from radiofrequency (RF) signal analysis, which allows attenuation estimation prior to envelope detection and may reduce the influence of system-dependent post-processing in certain implementations. In contrast, techniques such as TAI and USAT are commonly implemented using envelope-detected signals, which facilitates their integration into conventional ultrasound systems but may introduce additional variability related to device-specific processing pipelines (Bende et al., 2021; Park et al., 2022; Wu et al., 2024). Collectively, these techniques complement each other, but standardization of acquisition protocols and validation across vendors remains a key unmet need.

Overall, attenuation-based QUS techniques, including CAP, TAI, UGAP, USAT, and UDFF, demonstrate moderate to strong correlation with MRI-PDFF, which is widely regarded as the reference standard for noninvasive hepatic fat quantification. Reported correlation coefficients (r) range from 0.55 to 0.91, with AUC values between 0.82 and 0.96 for detecting ≥S1 steatosis (Li et al., 2024; Dag et al., 2025; Byenfeldt et al., 2026). UDFF, integrating both attenuation and backscatter measurements, exhibits the highest agreement with MRI-PDFF (AUC 0.90–0.91), providing quantitative liver fat estimates across a broad spectrum of steatosis severity (Xue et al., 2025). These findings highlight that while individual attenuation-based methods vary in accuracy and operator dependency, collectively they offer a reliable, noninvasive alternative to MRI-PDFF for clinical and research applications.

**Figure 4:**
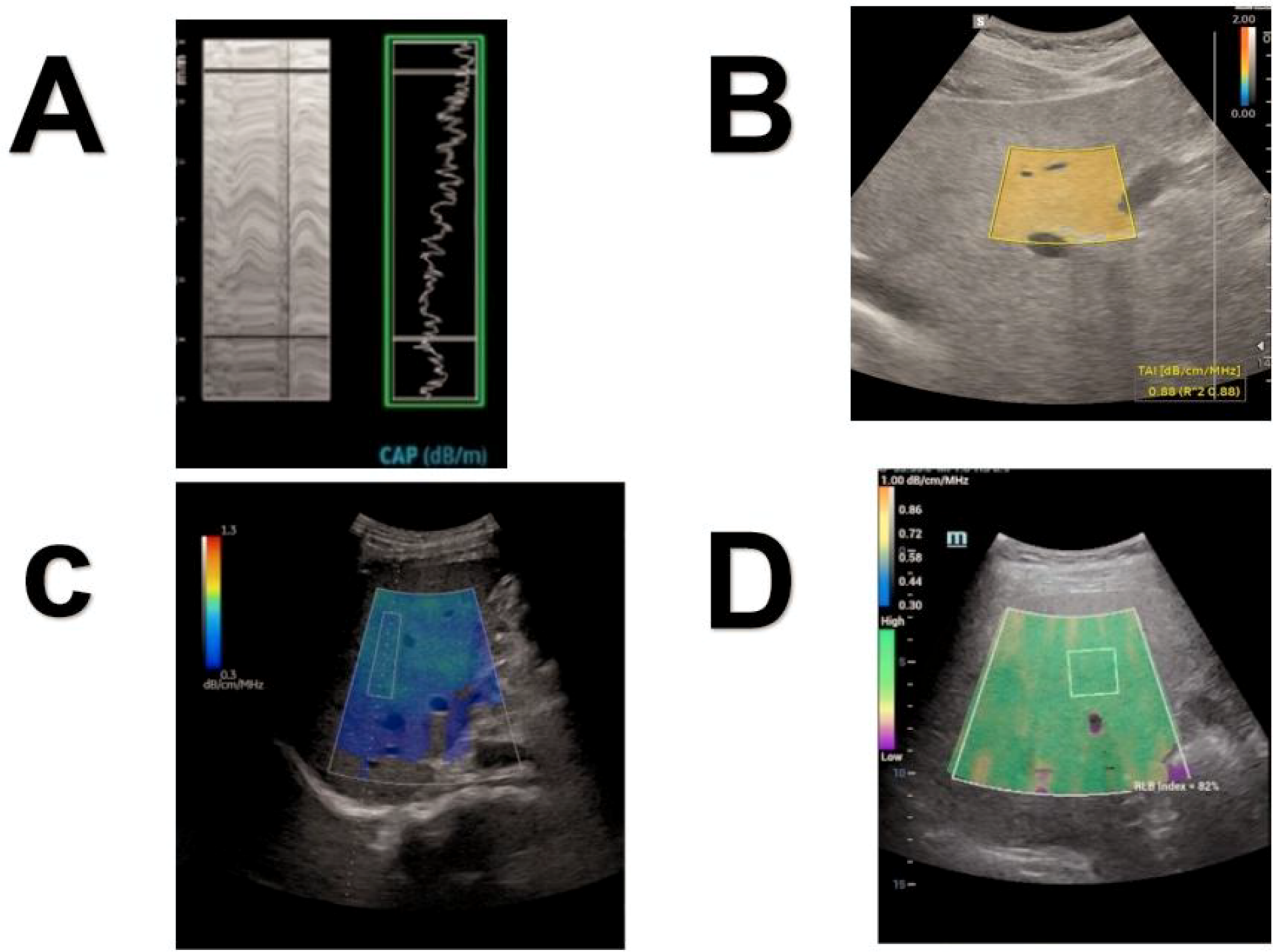
Representative ultrasound images of attenuation-based techniques for hepatic fat quantification. **(A)** CAP (Controlled Attenuation Parameter); **(B)** TAI (Tissue Attenuation Imaging); **(C)** UGAP (Ultrasound-Guided Attenuation Parameter); **(D)** USAT (Ultrasound Attenuation Analysis). Each modality estimates hepatic fat content by analyzing signal attenuation patterns with or without real-time B-mode image fusion.

As shown in **Figure 4**, attenuation-based techniques differ in visualization modes and image integration. CAP operates independently of B-mode imaging, whereas TAI, UGAP, and USAT offer real-time anatomical overlays.

## Backscatter-based techniques for microstructural tissue assessment

Backscatter-based techniques provide a unique perspective on liver microarchitecture. These approaches analyze the intensity, variability, and spatial distribution of ultrasound echoes reflected by hepatic parenchyma, which are influenced by the size, arrangement, and acoustic properties of cellular and extracellular structures. While some studies have reported correlations between backscatter parameters and fibrosis, these methods provide only indirect assessment of fibrotic changes and are less validated for fibrosis staging compared with elastography-based approaches. The principal backscatter techniques investigated in the context of MASLD include the Backscattering Coefficient (BSC), Tissue Scattering Imaging (TSI), and statistical approaches such as Nakagami imaging and Acoustic Structure Quantification (ASQ) (Zheng and Vaezy, 2010).

### Backscattering coefficient

BSC quantifies ultrasound energy scattered by liver microstructures, reflecting acoustic properties linked to steatosis and fibrosis (Lu et al., 1999; Gaitini et al., 2004). BSC reflects not only tissue microstructure but also the frequency-dependent scattering properties of acoustic scatterers, thereby providing a more comprehensive characterization of hepatic tissue composition. Small studies report significant correlation between BSC values and histologic steatosis grades (Bamber et al., 1981; Zagzebski et al., 1993; Paige et al., 2017).

Clinical use of BSC is limited by lack of calibration standards and sensitivity to beam angle and depth. Most applications remain experimental (Lin et al., 2015). With further development and validation, BSC may serve as a precise biomarker for early MASLD detection and integration into multiparametric QUS (Han et al., 2018).

### Tissue scattering imaging

TSI is a statistical backscatter method that models echo amplitude distributions (e.g., Nakagami, homodyned K) to capture microstructural heterogeneity from fat and fibrosis (Rónaszéki et al., 2022).It runs on standard ultrasound systems, enabling real-time visualization without extra hardware (Rónaszéki et al., 2022).TSI requires access to raw RF data for reliable analysis of backscatter characteristics, which is not routinely available on all ultrasound systems. Therefore, TSI is not universally applicable across standard machines, and its clinical use is currently limited to platforms providing RF data access.

TSI remains investigational, with limited standardization and commercial availability. Its accuracy is affected by ROI selection and operator variability. While early studies link TSI to histologic steatosis, broader validation and integration with other QUS metrics are needed for clinical use.

### Other statistical-based methods: Nakagami imaging and acoustic structure quantification

In addition to conventional BSC analysis, statistical approaches such as Nakagami imaging and Acoustic Structure Quantification (ASQ) assess liver parenchymal heterogeneity. Nakagami imaging models the echo envelope to estimate scatterer distribution, aiding early steatosis detection. ASQ analyzes backscatter histograms to infer structural abnormalities and is integrated into select commercial systems (e.g., Hitachi) (Oelze and Mamou, 2016; Son et al., 2016).

While these methods offer microstructural insights, their clinical utility in MASLD is limited. Nakagami imaging lacks standardized cut-offs and is sensitive to speckle noise; ASQ is no longer widely supported on modern platforms. Both remain research tools requiring further clinical validation (Son et al., 2016; Alvarenga et al., 2017).

**Figure 5:**
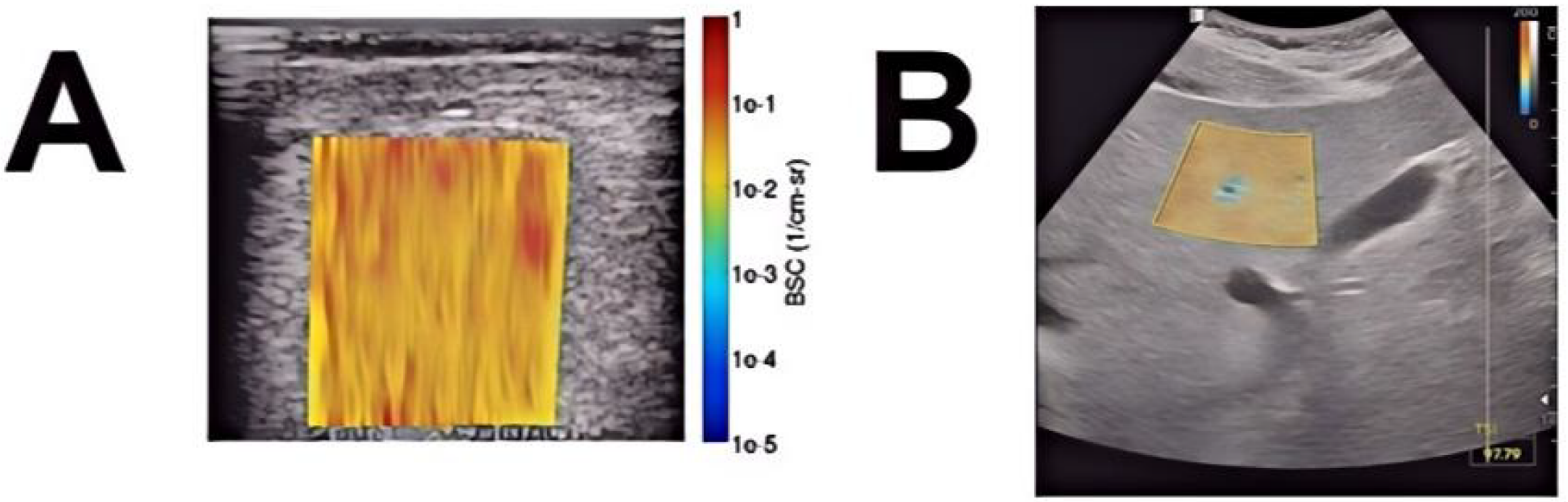
Representative images of backscatter- and grayscale-based techniques for liver characterization. **(A)** BSC; **(B)** TSI. These methods analyze echo intensity or grayscale ratios to reflect microstructural alterations linked to steatosis and fibrosis.

**Figure 5** illustrates representative imaging appearances of backscatter- and grayscale-based QUS techniques, which rely on echo amplitude and texture distribution to assess hepatic tissue composition.

### Speed of sound

SoS imaging estimates the acoustic propagation velocity through liver tissue, providing insight into its composition and structural integrity (Kumagai et al., 2014). In biological soft tissues, SoS varies with tissue density and compressibility: it decreases in lipid-rich regions and increases in fibrotic or collagen-rich areas (Ferraioli et al., 2021; Park et al., 2022). Importantly, SoS, like attenuation, is a fundamental acoustic parameter that reflects intrinsic tissue properties. Unlike stiffness-based metrics, SoS measurements are mainly sensitive to hepatic fat content; however, fibrotic remodeling and other tissue changes may also affect the measured sound speed, and thus interpretation of SoS should consider potential confounding effects. This distinction is essential to avoid misinterpreting SoS as an elasticity index (Imbault et al., 2017).

Recent ultrasound platforms have incorporated SoS measurement modules based on echo shift tracking, pulse-echo time-of-flight analysis, or sound speed tomography. Studies have shown that SoS decreases with increasing hepatic fat content, correlating inversely with histological steatosis and MRI-PDFF (Bonekamp et al., 2014; Nam et al., 2023). Additional studies in human and ex vivo liver samples have confirmed this relationship, demonstrating that higher liver fat content is associated with lower measured sound speed values (Benjamin et al., 2018).Compared to backscatter or attenuation metrics, SoS imaging offers better repeatability and has been reported to be less affected by superficial artifacts, including subcutaneous fat and rib shadowing, thereby improving its robustness in clinical application.

A key advantage of SoS-based QUS is its direct physical interpretability, enabling cross-vendor calibration and potential integration into multiparametric frameworks. In addition, SoS measurements can often be acquired simultaneously with attenuation or backscatter data, providing complementary parameters without increasing scan time (Park et al., 2022).

However, SoS imaging is not without limitations. Factors such as tissue anisotropy, phase aberration, and focal depth may introduce variability in measurements. Moreover, as with other emerging QUS modalities, standardization across platforms and clinical validation in large cohorts remain necessary for broader adoption (Lin et al., 2021).Nonetheless, SoS imaging holds promise as a quantitative and physically grounded tool for MASLD diagnosis, particularly in early-stage disease where subtle parenchymal changes may precede stiffness or attenuation shifts.

**Figure 6:**
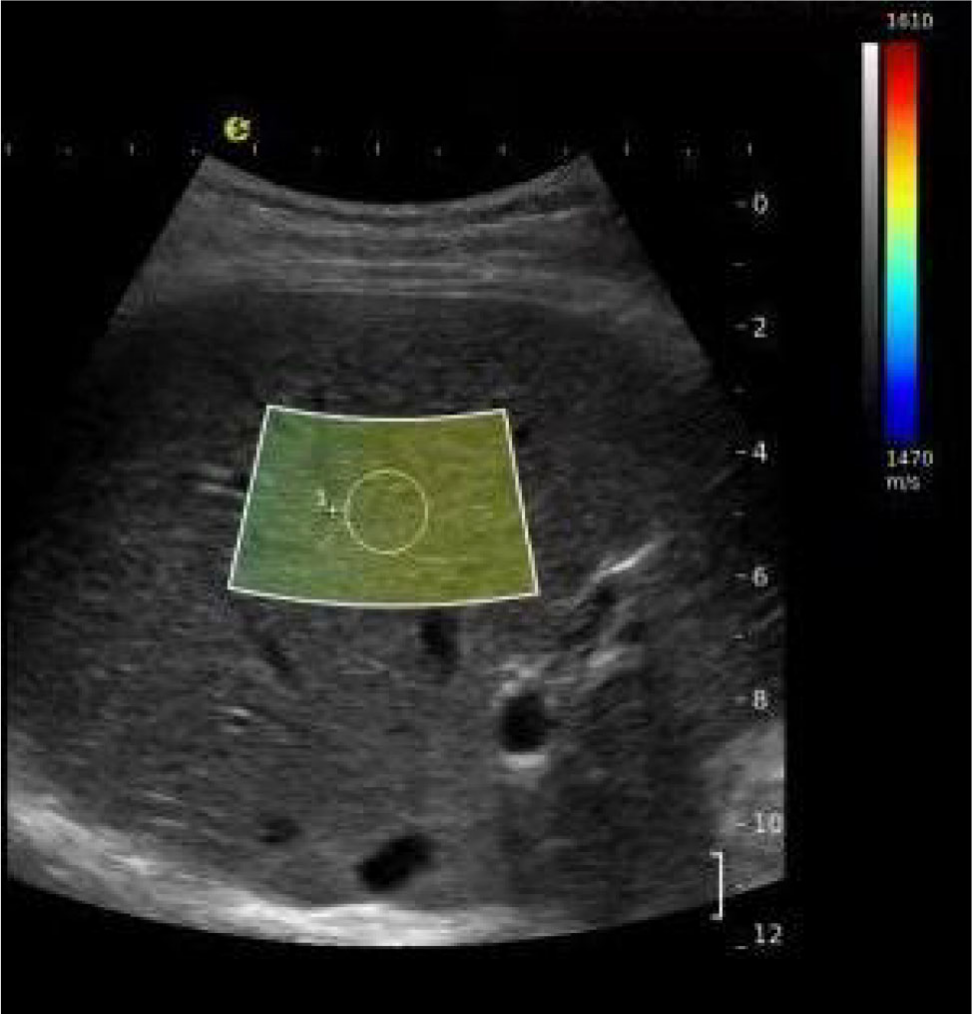
Representative SOS imaging for liver assessment. A color-coded map overlays the ROI, showing measured sound velocity (m/s), which reflects tissue composition and fat infiltration.

As illustrated in **Figure 6**, SOS imaging overlays liver sound velocity values on B-mode scans, enabling rapid and quantitative estimation of hepatic fat-related changes.

## Elasticity-based techniques for assessing fibrosis and inflammation

Liver stiffness, which reflects changes in the mechanical properties of tissue due to collagen deposition, edema, and inflammatory infiltration, is an important parameter in the noninvasive assessment of MASLD, particularly for evaluating fibrosis severity and disease progression. Elasticity-based QUS techniques rely on the propagation speed of shear waves through liver parenchyma, which increases proportionally with stiffness. These methods allow for dynamic assessment of fibrotic and inflammatory changes that underlie MASLD progression and prognosis.

### Transient elastography

TE is the earliest and most widely adopted method for liver stiffness quantification. It uses a mechanical vibrator to generate low-frequency shear waves and measures their velocity using pulse-echo ultrasound, providing a single-point stiffness estimate typically expressed in kilopascals (kPa). Its diagnostic performance for detecting significant fibrosis in MASLD has been extensively validated, with multiple studies reporting AUCs exceeding 0.85 (Kaewdech et al., 2024; Mohanty et al., 2025). Recent multicenter studies and meta-analyses further support TE as a reliable noninvasive tool for fibrosis staging and risk stratification in MASLD patients (Eid et al., 2025; Masrour et al., 2025).

Despite its simplicity and speed, TE lacks real-time image guidance, making it prone to sampling errors, especially in obese patients, or those with narrow intercostal spaces or ascites. Furthermore, it cannot assess parenchymal heterogeneity and is limited to the right lobe of the liver.

### Shear wave elastography

SWE includes both point shear wave elastography (pSWE) and two-dimensional shear wave elastography (2D-SWE), both of which use acoustic radiation force to generate shear waves within the liver parenchyma (Barr et al., 2016). The shear wave velocity, or its converted elastic modulus, is calculated based on the time taken for the wave to travel through the tissue (Cassinotto et al., 2016; Ozturk et al., 2020; Luo et al., 2022).

pSWE allows single-point measurements at operator-selected ROIs under real-time B-mode guidance, reducing the risk of sampling large vessels or artifacts (Bota et al., 2012; EASL-ALEH Clinical Practice Guidelines, 2015; Castera et al., 2019; Gatos et al., 2020). 2D-SWE further expands this approach by providing a color-coded stiffness map over a wider field of view, enabling visualization of tissue heterogeneity and improving the reliability of fibrosis assessment.

Compared to TE, SWE offers image-guided targeting, higher reproducibility, and the flexibility to scan multiple liver segments. However, inter-vendor variability, dependency on probe orientation, and susceptibility to inflammation-related confounders remain limitations (Garcovich et al., 2017; Wang et al., 2020).

### Shear wave dispersion

SWD imaging measures the frequency dependence of shear wave velocity, offering insight into the tissue’s viscoelastic properties, particularly viscosityn (Sugimoto et al., 2020; Zhang et al., 2022).

Increased dispersion slope is thought to reflect inflammation-related changes such as cellular edema, necrosis, and matrix disorganization (Kaewdech et al., 2024; Mohanty et al., 2025). Unlike elasticity alone, viscosity-related parameters are hypothesized to be more closely associated with necroinflammatory activity.

Techniques such as Vi.PLUS extract dispersion metrics during standard 2D-SWE acquisition, adding no extra scan time. Several clinical studies have demonstrated that SWD parameters are significantly associated with histological inflammatory activity scores and may improve the discrimination of steatohepatitis (MASH) from simple steatosis (Zhang et al., 2017; Gao et al., 2022).These findings support the role of SWD as a complementary biomarker to elasticity, particularly for characterizing disease activity in MASLD.

Despite its promise, SWD remains under investigation. Its sensitivity to both inflammation and fibrosis may complicate interpretation, and further technical standardization and validation in large cohorts are required before routine clinical implementation.

### Real-time tissue elastography

RTE, or strain elastography, evaluates tissue deformation in response to manual or physiological compression. By comparing relative tissue displacement, RTE generates a color map or strain ratio that reflects stiffness (Morikawa et al., 2011).

Although RTE is more operator-dependent and lacks absolute stiffness values, it is valuable in certain scenarios, such as assessing superficial lesions or guiding liver biopsy. Although strain elastography has been investigated for liver fibrosis assessment in chronic liver diseases, the available evidence specifically addressing MASLD remains limited. Early exploratory studies have suggested that RTE-derived strain patterns or strain ratios may correlate with histological fibrosis stages, but these observations are based on relatively small cohorts and heterogeneous study designs. Consequently, the clinical role of RTE in MASLD is currently considered supplementary and investigational compared with shear-wave–based elastography techniques (Colombo et al., 2012; Orlacchio et al., 2012; Schenk et al., 2014; Grgurevic et al., 2015).Unlike shear-wave–based techniques, RTE provides relative strain information rather than absolute stiffness measurements, and interpretation is typically based on qualitative patterns or strain ratios.

However, its dependence on external compression, limited reproducibility, and lack of standardized thresholds restrict its routine use. As newer techniques evolve, RTE has become less favored but may still contribute in multimodal or AI-augmented frameworks.

For fibrosis assessment, elasticity-based QUS methods, particularly transient elastography (TE) and shear-wave elastography (SWE), have demonstrated comparable diagnostic performance to MR Elastography (MRE) for detecting clinically significant fibrosis (F2–F4), with reported AUC values ranging from 0.81 to 0.89 and similar sensitivity and specificity (Castera et al., 2019; Ozturk et al., 2022). Emerging techniques, including shear-wave dispersion (SWD) and real-time tissue elastography (RTE), provide complementary information on viscoelastic properties, although their validation against MRE is currently limited to smaller cohort studies. Together, these methods offer a practical, noninvasive alternative to MRE for fibrosis staging in MASLD, while highlighting the need for further standardization and multicenter validation (Rhee et al., 2025).

**Figure 7:**
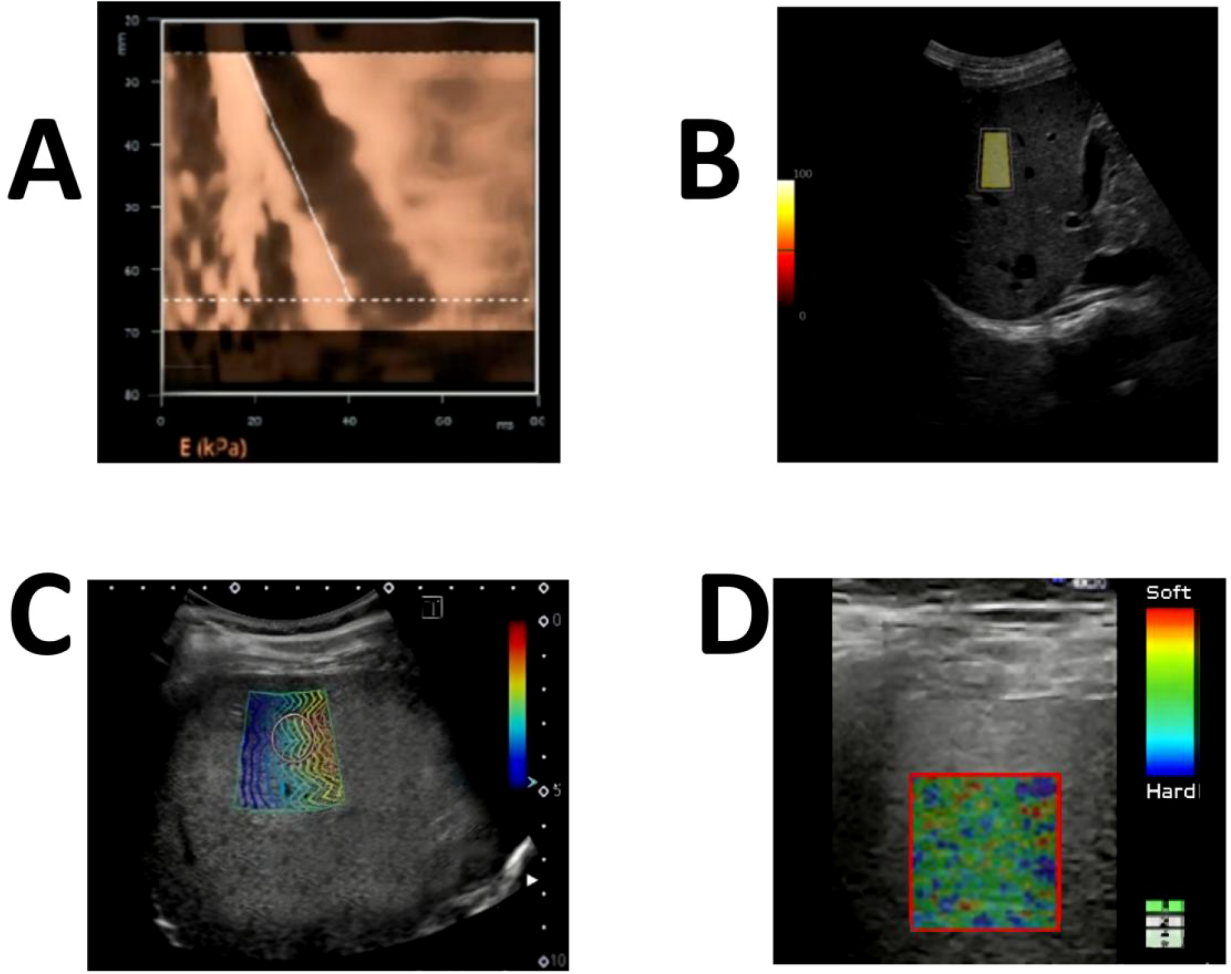
Representative elastographic techniques used for liver stiffness and viscoelasticity assessment. **(A)** Transient Elastography (TE), providing a single-point liver stiffness measurement typically expressed in kilopascals (kPa). **(B)** Two-dimensional Shear Wave Elastography (2D-SWE), displaying a color-coded elasticity map that quantitatively represents tissue stiffness within the selected region of interest. **(C)** Shear Wave Dispersion (SWD), illustrating frequency-dependent shear-wave propagation associated with tissue viscoelastic properties and necroinflammatory activity. **(D)** Real-time Tissue Elastography (RTE), demonstrating a strain-based color map reflecting relative tissue deformation under compression. In RTE imaging, the displayed scale represents relative strain distribution rather than absolute stiffness values, and interpretation is typically based on qualitative patterns or semi-quantitative strain ratios within the region of interest.

**Figure 7**. Visual and functional comparison of elasticity-based techniques—TE, SWE, SWD, and RTE—illustrates their respective strengths in assessing liver stiffness and viscoelastic properties.

## Microvascular imaging techniques

The progression of MASLD involves not only steatosis and fibrosis, but also subtle yet significant alterations in hepatic microcirculation. Sinusoidal capillarization, inflammatory infiltration, and extracellular matrix deposition can impair normal perfusion patterns, which may precede overt fibrosis or steatohepatitis. Microvascular imaging has emerged as a promising noninvasive approach to capture these early pathophysiological changes. Recent advances in ultrasound technology, particularly Superb Microvascular Imaging (SMI) and Ultra Microvascular Analysis (UMA), enable detailed visualization of low-velocity hepatic blood flow without contrast agents, offering complementary hemodynamic information alongside conventional and elastographic imaging.

### Superb microvascular imaging

SMI is a Doppler-based technique that employs advanced clutter suppression algorithms to isolate microvascular flow signals from surrounding motion artifacts. Unlike conventional color Doppler, SMI can visualize vessels with diameters below 0.1 mm and detect blood flow velocities as low as a few millimeters per second, making it especially suited for assessing the sinusoidal network (Liu et al., 2017).

Emerging studies have explored the application of SMI for evaluating alterations in intrahepatic microvascular architecture. Preliminary investigations suggest that SMI-derived vascular patterns may reflect changes in sinusoidal perfusion associated with hepatic steatosis and inflammatory activity. For example, reductions in vascular branching complexity and heterogeneous perfusion signals have been reported in patients with steatohepatitis compared with simple steatosis. These findings indicate that SMI may provide complementary hemodynamic information beyond conventional grayscale imaging and elastography, although the current evidence remains limited and further clinical validation is required (Aziz et al., 2022; Gao et al., 2022).

While promising, SMI is still limited by vendor-specific implementation, lack of quantitative flow metrics, and operator dependency. Nonetheless, it represents an important step toward noninvasive assessment of hepatic microvascular integrity.

### Ultra microvascular imaging

UMA builds upon the principles of SMI but integrates frame-to-frame signal tracking and statistical noise modeling to enhance microvessel detection sensitivity. UMA enables dynamic assessment of vascular complexity, perfusion homogeneity, and vessel density using high-definition B-mode sequences alone (Lee et al., 2016).

Preliminary investigations have explored the potential of ultra-microvascular imaging techniques to characterize subtle alterations in hepatic microvascular patterns in metabolic fatty liver disease. These studies suggest that quantitative descriptors of vascular texture and perfusion uniformity may differ between patients with simple steatosis and those with steatohepatitis or early fibrotic changes; however, the available data remain limited and largely exploratory (Lee et al., 2016; Mu et al., 2019). Because UMA does not require Doppler acquisition or contrast agents, it offers a low-cost and radiation-free alternative for tracking microvascular remodeling (Nam et al., 2022).

However, like SMI, UMA remains primarily a research tool. The lack of standardized interpretation protocols and limited longitudinal data have constrained its clinical adoption. Future studies incorporating histological validation and AI-driven vessel segmentation may enhance its diagnostic power and reproducibility.

Microvascular imaging techniques, including SMI and UMA, provide complementary hemodynamic information and early detection of perfusion alterations, but are not directly benchmarked against MRI-PDFF or MR Elastography, as these modalities primarily assess fat and fibrosis.

**Figure 8:**
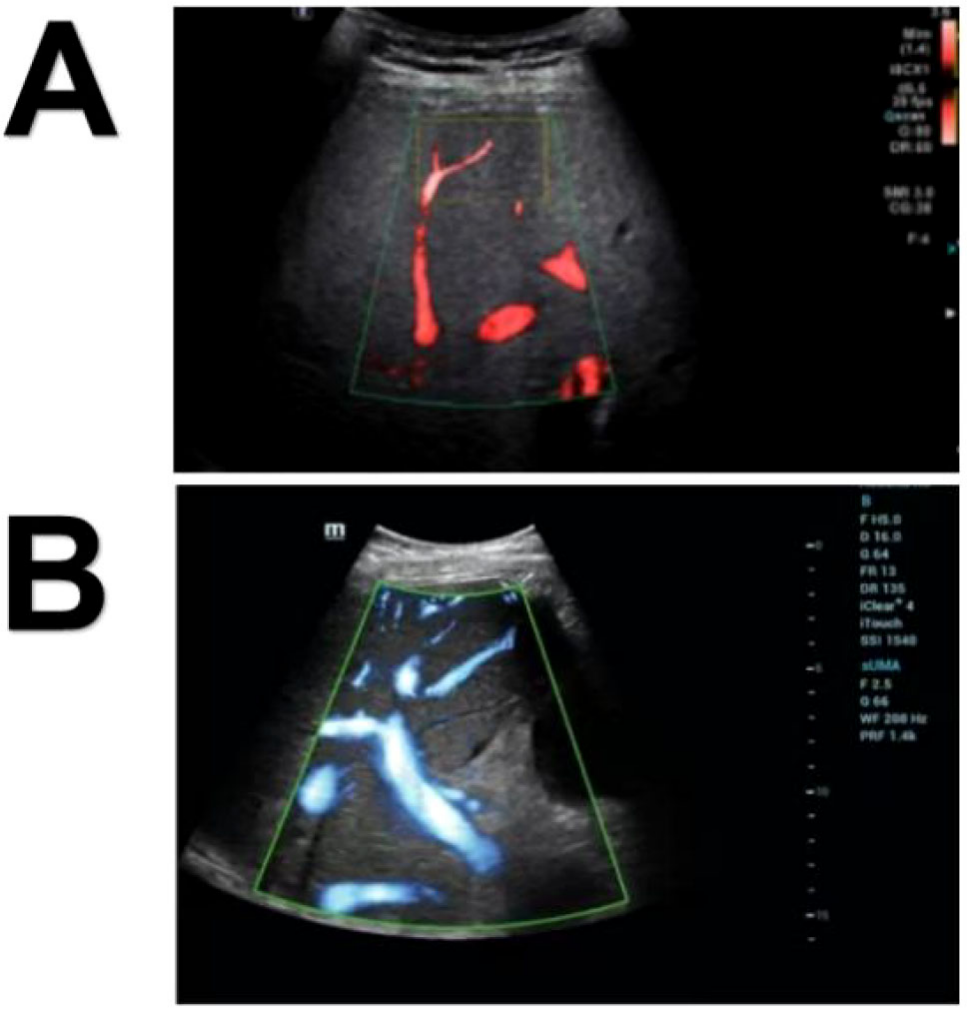
Representative microvascular imaging techniques for liver assessment. **(A)** Superb Microvascular Imaging (SMI); **(B)** Ultra Microvascular Analysis (UMA). Both visualize slow-flow hepatic vasculature without contrast agents, aiding non-invasive perfusion evaluation.

As illustrated in **Figure 8**, SMI and UMA enable non-contrast microvascular visualization, supporting the assessment of hepatic perfusion and inflammation in MASLD.

## Multiparametric and hybrid techniques

UDFF represents one of the most clinically advanced multiparametric QUS techniques for hepatic fat quantification. Developed to mimic the PDFF obtained via MRI, UDFF estimates liver fat content by integrating both ultrasound attenuation and backscatter coefficients into a proprietary algorithm (Dominik et al., 2025). It is the first and, to date, the only ultrasound-based method to receive FDA approval for the quantification of hepatic steatosis (Bozic et al., 2022).

By combining biophysically distinct parameters, UDFF enhances the reliability of liver fat assessment compared to attenuation-based techniques alone. Attenuation reflects energy loss due to fat scattering and absorption, while backscatter captures microstructural interference and echogenicity changes caused by lipid droplets (Gao et al., 2021). The integration of these measurements reduces susceptibility to confounders such as liver fibrosis, body habitus, and scanning angle, thereby improving diagnostic accuracy across a broader patient spectrum.

Studies have shown that UDFF correlates strongly with MRI-PDFF and histological steatosis grading, and performs well in differentiating mild from moderate-to-severe steatosis (Qi et al., 2025). Importantly, UDFF acquisition can be seamlessly integrated into standard abdominal ultrasound exams, offering a cost-effective and scalable solution for hepatic fat screening in routine practice.

Despite its clinical advantages, challenges remain regarding inter-system reproducibility, vendor lock-in, and interpretation thresholds across diverse populations. Nevertheless, UDFF exemplifies the clinical potential of multiparametric QUS and serves as a prototype for future hybrid imaging technologies aimed at noninvasive metabolic liver disease characterization.

**Figure 9:**
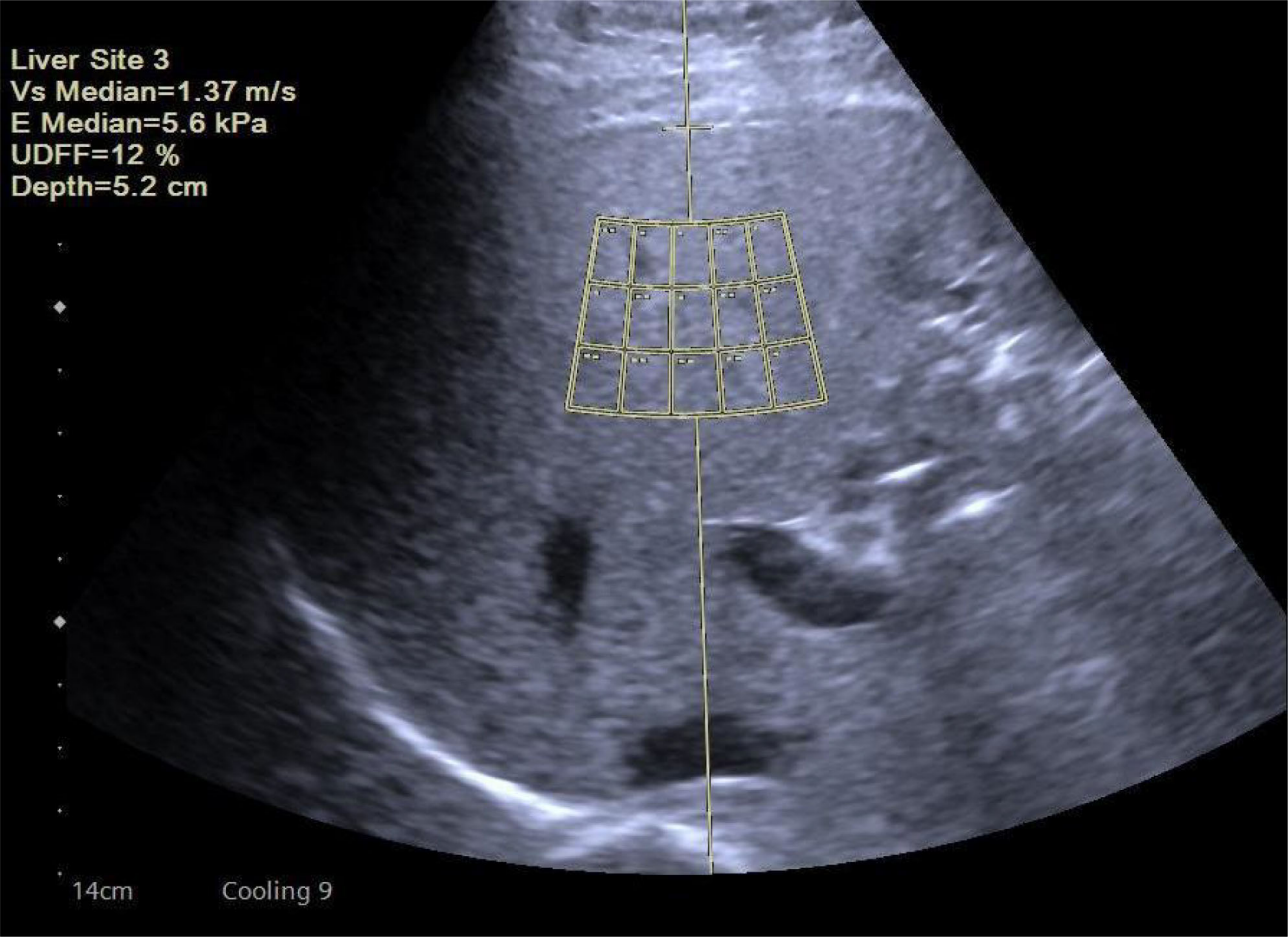
Representative UDFF image showing grid-based ROI with liver fat percentage, allowing real-time, quantitative assessment of hepatic steatosis.

As shown in **Figure 9**, UDFF offers real-time estimation of hepatic fat percentage by combining attenuation and backscatter signals across multiple ROIs.

Beyond UDFF, commercial systems increasingly adopt multiparametric QUS. Philips ElastPQ integrates SWE, AI, and B-mode imaging, while Samsung’s Liver Toolbox acquires TAI, SWI, and backscatter data concurrently (Mare et al., 2017). These platforms streamline workflow and enable simultaneous evaluation of fat, stiffness, and tissue heterogeneity—critical for characterizing MASLD patients with overlapping features (Ferraioli et al., 2018).

Emerging AI-assisted frameworks integrate QUS features (e.g., CAP, SWE, SOS) into neural or statistical models to predict histological subtypes. Though experimental, these approaches show promise in surpassing single-parameter methods, especially in borderline or diagnostically ambiguous MASLD cases (Yan et al., 2024).

A comparative overview of these attenuation-based techniques—including CAP, TAI, UGAP, and USAT—is summarized in **Table 1**.

**Table 1:** Comparative summary of key quantitative ultrasound (QUS) techniques for the assessment of MASLD.

Methods	Technique	Principle	Primary target	Advantages	Limitations
Steatosis-focused methods	HRI	Grayscale echo ratio (liver/kidney)	Moderate-to-severe steatosis	Simple, widely compatible, primary-care friendly	Sensitive to machine settings and renal disease; poor for mild steatosis
	CAP	Attenuation (Transient Elastography)	Steatosis	Widely available, quick, paired with fibrosis assessment	Affected by BMI, no 2D guidance
	TAI	Attenuation (2D Ultrasound, envelope-based)	Steatosis	Real-time, operator-guided, good for S1	Depth limits, affected by ascites
	UDFF	Dual-parameter: Attenuation + Backscatter	Steatosis (Quantitative %)	MRI-correlated, depth-independent, ROI tracking, real-time	Vendor lock-in, no unified cut-offs, limited multicenter validation
	USAT	Wide-field Attenuation Analysis (envelope-based)	Steatosis	Good repeatability in early studies, reduced sampling variability	Limited multicenter validation, no guideline thresholds, cross-platform comparability unknown
	UGAP	Image-guided attenuation coefficient estimation (vendor-dependent implementation)	Steatosis	Standardized acquisition, less influenced by post-processing	Limited availability; moderate correlation with MRI-PDFF only
	BSC	Backscatter Coefficient (frequency-dependent scattering)	Steatosis	Correlates with steatosis; research-grade repeatability	Needs post-processing; no standard thresholds; limited clinical validation
	TSI	Backscatter/texture from RF data (e.g., homodyned-K/Nakagami)	Early steatosis	Micro-architectural insight, potential for S1 detection	Requires RF access; limited availability; device variation
	ASQ	Statistical texture analysis (historical, vendor-specific)	Steatosis	Semi-automated; easy to compute	Largely obsolete; sensitive to gain/depth; limited clinical utility
	Nakagami Imaging	Envelope statistics of echo amplitudes	Microstructural steatosis change	Sensitive to early fat change; less gain-dependent in research	Research-stage; no thresholds; not clinically implemented
	SoS	Sound-speed measurement (fundamental acoustic property)	Steatosis	Good repeatability; less affected by rib shadowing than attenuation; screening-friendly	Standardization needed; performance in obesity under study; can be affected by tissue heterogeneity
Fibrosis-focused methods	SWE	Shear-wave velocity	Fibrosis	Widely adopted, reproducible, validated vs biopsy	Lower sensitivity for early fibrosis; operator/probe dependence
	SWD	Shear-wave dispersion (viscoelasticity)	Necroinflammation/Fibrosis	Associated with necroinflammatory activity; viscoelastic insight	Protocol-sensitive; influenced by stiffness extremes; requires further standardization
	RTE	Strain elastography (relative tissue deformation)	Exploratory fibrosis assessment/tissue stiffness pattern	Simple visualization of relative strain distribution; may provide qualitative stiffness information	Operator-dependent; relative measurement without absolute stiffness values; limited validation in MASLD
Other/Microvascular/AI	SMI/UMA	Low-velocity flow imaging (microvasculature)	Perfusion	Non-contrast visualization of slow flow	Operator-dependent; limited quantitative metrics and evidence
	AI-Assisted	Deep-learning/radiomics on QUS	Steatosis/Fibrosis (± activity)	Automated, scalable; reduces variability; enables multiparametric fusion	Needs large, well-annotated datasets; external validation & regulatory hurdles

This matrix contrasts methods in terms of their physical principles, target pathology (steatosis, fibrosis, inflammation, or perfusion), diagnostic advantages, and key limitations. It illustrates the diversity of modalities across biophysical domains and highlights complementary roles among attenuation, elasticity, backscatter, and AI-based methods.

## AI in QUS for MASLD: challenges and opportunities

AI, particularly deep learning, has rapidly expanded its role in QUS for the evaluation of MASLD. AI has been extensively explored for applications such as automated image interpretation, feature extraction, and disease classification. These advancements help mitigate operator dependency, improve consistency, and enhance the diagnostic accuracy of QUS systems (Rhyou and Yoo, 2021).

Key applications include automated liver segmentation using Convolutional Neural Networks (CNNs), which improve the consistency of ROI placement across modalities like SWE, ATI, and UDFF. CNNs have also been trained to classify liver steatosis and fibrosis, achieving diagnostic accuracies comparable to, or surpassing, traditional QUS and MRI-based methods. Radiomics further enhances AI’s predictive power by extracting high-dimensional features from QUS images, enabling the detection of subtle tissue heterogeneity (Li et al., 2022).

Recent advances in artificial intelligence (AI) have enabled more sophisticated analysis of quantitative ultrasound (QUS) data for MASLD assessment. Convolutional Neural Networks (CNNs) and radiomics-based models have been applied to raw or processed QUS images, integrating parameters such as attenuation coefficients, shear-wave elastography maps, backscatter features, and sound-speed values. Models are typically trained on annotated datasets with histological or MRI-based references, employing cross-validation or hold-out strategies to evaluate performance. Reported diagnostic accuracies for AI-assisted QUS range from AUC 0.90 to 0.93 for steatosis and fibrosis classification, surpassing single-parameter QUS metrics and demonstrating potential for multiparametric integration.

Despite these promising developments, several challenges continue to limit the widespread clinical adoption of AI-assisted QUS in MASLD assessment. Many currently available models are trained on relatively small or single-center datasets, which raises concerns about their generalizability across different patient populations and imaging platforms. In addition, variability in ultrasound acquisition protocols and hardware configurations across vendors may affect the reproducibility of AI-based analyses. Another important limitation relates to the interpretability of deep learning models, which are often perceived as “black-box” systems and may therefore hinder clinical trust and regulatory acceptance. Addressing these challenges will require larger multicenter datasets, improved cross-platform standardization, and the development of explainable AI frameworks that enhance transparency and clinical usability (Tahmasebi et al., 2023).

Furthermore, clinical integration faces hurdles such as regulatory approval, data privacy, and the need for AI systems to fit into existing workflows. AI tools must be validated through multi-center clinical trials and demonstrate user-friendliness, providing actionable insights within clinical time constraints (Li et al., 2025).

In conclusion, AI-enhanced QUS holds significant promise for improving the non-invasive assessment of MASLD. However, addressing challenges related to data quality, model generalizability, and clinical integration will be key to realizing its potential in personalized medicine for liver disease.

**Table 2** summarizes the diagnostic performance of quantitative ultrasound (QUS) techniques for MASLD. Attenuation-based methods, including CAP, TAI, UGAP, USAT, and UDFF, show AUC values of 0.82–0.91 for detecting ≥S1 steatosis, with correlation coefficients (r) of 0.55–0.91 versus MRI-PDFF. Elasticity-based methods (TE, SWE) demonstrate comparable performance to MR Elastography for significant fibrosis (F2–F4), with AUCs ranging from 0.81–0.89 and similar sensitivity/specificity. Emerging techniques, including SWD, RTE, and microvascular imaging (SMI/UMA), provide complementary information but are primarily investigational and require further validation. AI-based multiparametric models achieve AUCs up to 0.93, integrating multiple QUS parameters to enhance diagnostic accuracy. This consolidated overview provides quantitative metrics that complement the narrative discussion and enable direct comparison across modalities.

**Table 2:** Diagnostic performance of QUS techniques in MASLD.

Methods	QUS technique	Target	AUC/ performance	Sensitivity/ specificity	Cut-offs/ comments	Benchmark vs MRI-PDFF / MRE
Steatosis	HRI (Hepatorenal Index)	Steatosis	0.80–0.88	80–85% / 75–85%	Semi-quantitative grayscale index; compares liver and kidney echogenicity; operator-dependent; moderate diagnostic performance for moderate-to-severe steatosis	Moderate correlation with MRI-based fat quantification reported
	CAP (FibroScan)	Steatosis≥ S1	0.82–0.88	80–85% / 75–85%	Cut-offs:S1 ~268 dB/m, S2 ~280 dB/m;blind acquisition	r = 0.60–0.82; AUC 0.82–0.88 vs MRI-PDFF
	TAI	Steatosis≥ S1–S3	0.84–0.88 (≥ S2:0.84 [0.76–0.90])	80–85% / 78–98%	Cut-off: AC-TAI>0.884 dB/cm/MHz;operator-guided ROI	AUC 0.84–0.88 vs MRI-PDFF
	UGAP	Steatosis≥ S2	0.828 (0.739–0.896)	67–76% / 80–87%	Cut-off:0.67–0.74 dB/cm/MHz;Image-guided; vendor-dependent	r = 0.55–0.65 vs MRI-PDFF
	USAT	Steatosis (all grades)	0.96 (0.93–0.99)	93.6% / 95.5%	Grade AUCs: S1 0.86, S2 0.81, S3 0.87;Wide-field mapping; envelope-based	AUC 0.86–0.87 vs MRI-PDFF
	UDFF	Steatosis ≥ S1	0.90–0.91	87–94% / 60–94%	Combined attenuation+ backscatter;FDA-approved	AUC 0.90–0.91 vs MRI-PDFF
	TSI	Steatosis ≥ 5% MRI-PDFF	0.964	85.4% / 97.4%	Score >91.2;RF-based; experimental	Directly evaluated against MRI-PDFF in a comparative study
	BSC	Steatosis	Increasing trend with steatosis	—	Preliminary studies; no AUC reported;Research-stage; limited clinical validation	Comparative studies with MRI-based fat quantification have been reported, but evidence remains limited
	Nakagami Imaging	Steatosis	r = 0.84–0.86 with fat content	—	Research stage;Experimental; microstructural assessment	—
	ASQ	Steatosis (historical)	—	—	Obsolete method;research-only	—
	Speed-of-Sound (SoS)	Steatosis	—	—	Physical parameter, lacking clinical AUC; Fundamental parameter; limited clinical validation	Early comparative studies suggest agreement with MRI-PDFF–defined steatosis trends, but robust diagnostic thresholds are not yet established
Fibrosis	2D-SWE	Fibrosis ≥ F2 / advanced	0.81–0.86	80–86% / 64–76%	Cut-offs ~5.85–6.75 kPa;Image-guided; reproducible	Comparable to MRE (AUC 0.81–0.86)
	TE / VCTE	Fibrosis ≥ F2–F4	0.85–0.89 (F2)	76–89% / 63–82%	Meta-analysis derived;Single-point measurement; widely adopted	Comparable to MRE (AUC 0.85–0.89)
	SWD	Inflammation / viscosity	—	—	Research-phase shear viscosity technique;Experimental; viscoelastic insights	Limited MRE validation
Microvascular / Perfusion	SMI / UMA	Inflammation / MASH	—	—	Exploratory phase only; non-contrast flow visualization	Not benchmarked
AI / Multiparametric	AI-based QUS	Steatosis / MASH staging	AUC up to 0.90–0.93	—	Multi-parametric model fusion;dataset-limited; generalizability issues	Multi-parametric fusion

**Figure 10:**
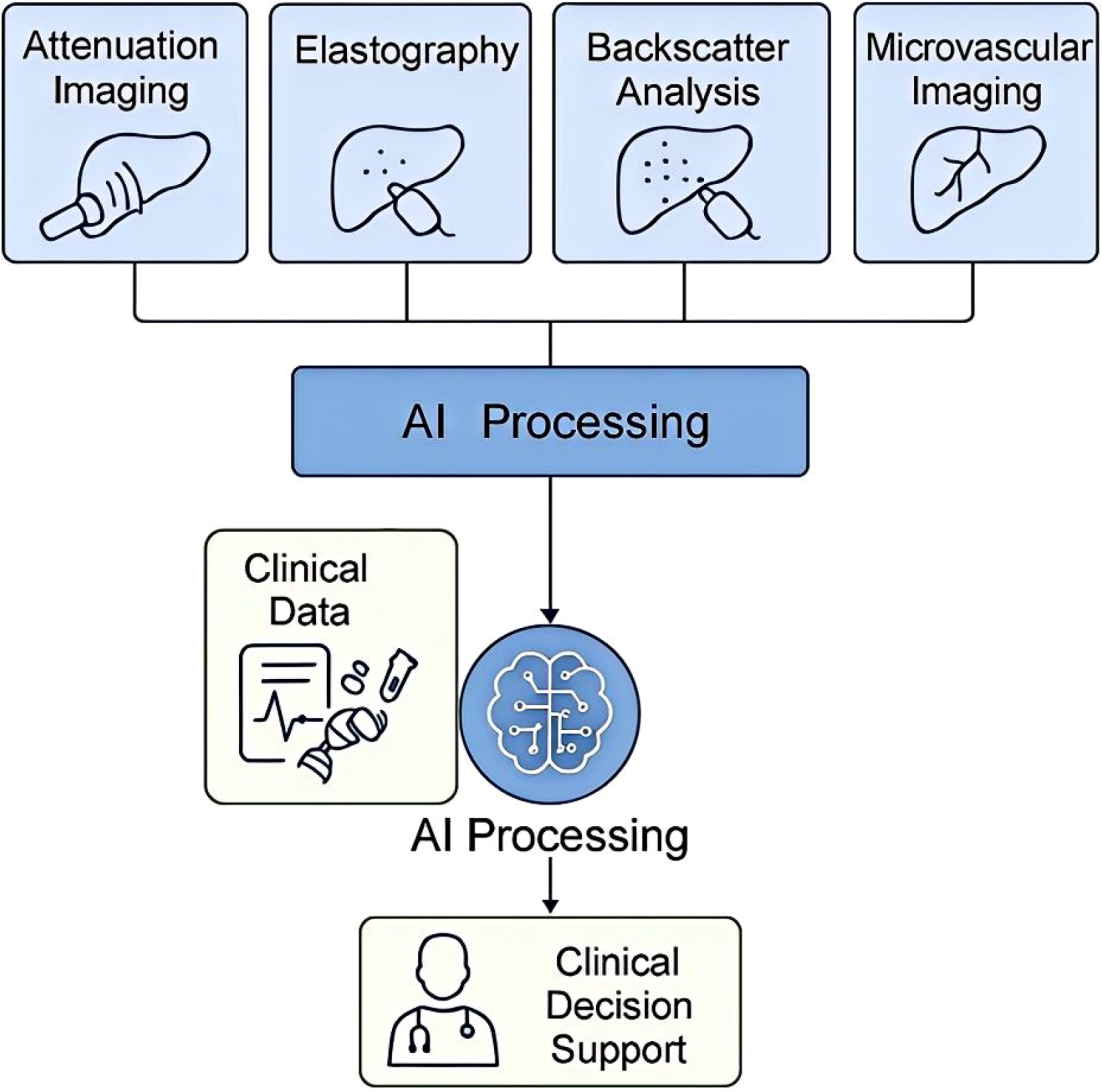
Workflow of AI-assisted QUS analysis for MASLD. Multiparametric QUS inputs (attenuation, elastography, backscatter, vascularity) are integrated with clinical data for diagnostic decision support.

As depicted in **Figure 10**, integrating QUS outputs with clinical data through AI pipelines enables more consistent and data-driven diagnostic decision-making in MASLD.

### Challenges and future perspectives

Despite significant advances in QUS technologies for the noninvasive assessment of MASLD, several challenges continue to hinder their widespread clinical adoption. First and foremost is the lack of standardization across vendors, protocols, and parameter units. QUS measurements such as liver stiffness, attenuation, and backscatter are highly dependent on machine settings, probe orientation, and operator expertise. The absence of universally accepted cutoff values for steatosis, inflammation, and fibrosis further complicates interpretation and limits generalizability across populations (Chan et al., 2014).

Addressing these challenges requires unified standardization strategies. These include cross-platform calibration using tissue-mimicking phantoms or reference materials, standardized acquisition protocols (ROI placement, measurement depth, transducer frequency, number of repetitions), and harmonized cut-offs for steatosis and fibrosis. Multi-center validation is essential to ensure reproducibility and generalizability across populations.

Furthermore, multi-center validation studies are necessary to confirm the robustness and generalizability of QUS biomarkers in diverse patient populations. Such studies help identify device-specific biases and population-dependent factors, ultimately supporting broader clinical adoption.

These strategies are in line with recommendations from international consensus guidelines, including the WFUMB Guidelines on Liver Multiparametric Ultrasound (Ferraioli et al., 2024a; Ferraioli et al., 2024b), which emphasize standardized measurement procedures, cross-vendor comparability, and reproducible data acquisition. Together, cross-platform calibration, protocol harmonization, threshold standardization, and multi-center validation form a comprehensive framework to address vendor heterogeneity and facilitate reliable implementation of QUS in clinical practice. Future work should focus on integrating these strategies with AI-assisted QUS models to further enhance diagnostic accuracy and allow scalable deployment across diverse clinical settings.

Biological heterogeneity and patient-related factors, including disease stage, BMI, subcutaneous fat thickness, and comorbidities, influence QUS measurements such as stiffness, dispersion, and attenuation. These variables complicate the interpretation of liver fat content and stiffness, particularly in early-stage MASLD (Tada et al., 2019; Thiele et al., 2020; Heilani et al., 2024).

Technical limitations are also a barrier. Variations in probe frequency, angle of insonation, and system architecture contribute to inconsistencies across devices. Techniques requiring RF data (e.g., UGAP, TSI) are not universally available across ultrasound systems, limiting their broader clinical adoption (Song et al., 2020).

Study design limitations are also prevalent. Many studies are single-center, retrospective, and small, often relying on biopsy as the reference standard, which introduces bias toward advanced disease. Publication and reporting biases may further skew QUS performance assessments.

Moreover, although multiparametric and AI-enhanced QUS frameworks offer exciting opportunities, their clinical integration remains in its infancy. Recent international consensus guidelines, including the WFUMB Guidelines on Liver Multiparametric Ultrasound and liver fat quantification (Ferraioli et al., 2024a; Ferraioli et al., 2024b) and the EASL-EASD-EASO Clinical Practice Guidelines on MASLD management (EASL-EASD-EASO, 2024), provide important recommendations for standardized liver fat assessment and clinical management, supporting the integration of multiparametric QUS approaches. Issues such as model interpretability, data privacy, regulatory approval, and cross-platform validation must be addressed before these tools can be routinely implemented in real-world settings. Technical barriers persist as well. For example, QUS signal quality may be degraded in patients with obesity, ascites, or deep-seated liver parenchyma. Microvascular and radiomic techniques, while promising, require advanced hardware, specialized training, and further clinical validation.

Collectively, while quantitative ultrasound (QUS) provides valuable non-invasive assessment of MASLD, each modality has inherent limitations that should be considered. Attenuation-based techniques, including CAP, TAI, UGAP, USAT, and UDFF, are influenced by patient factors such as obesity or narrow intercostal windows and by operator-dependent ROI placement, and thresholds for steatosis grading vary across devices and populations. Elasticity-based methods (TE, SWE, SWD, RTE) are affected by inflammation, congestion, probe orientation, and cross-vendor reproducibility limitations, while emerging techniques such as SWD and RTE remain primarily investigational. Backscatter-based approaches, including BSC, TSI, Nakagami, and ASQ, provide insight into liver microstructure but only indirectly assess fibrosis and often require access to raw RF data, limiting clinical applicability. Microvascular imaging techniques (SMI, UMA) are largely exploratory, operator-dependent, and lack standardized interpretation protocols. AI-assisted QUS models demonstrate promising diagnostic performance but are limited by small datasets, cross-platform generalizability, and interpretability challenges. Awareness of these limitations is essential for accurate interpretation of QUS metrics and for planning robust clinical implementation.

Looking forward, future efforts should prioritize the harmonization of acquisition protocols, the development of large multicenter datasets, and the establishment of reference standards for each QUS parameter. Integration with AI, multimodal imaging (e.g., MRI, elastography, CEUS), and histological validation cohorts will be essential to refine diagnostic algorithms and support personalized disease staging. Ultimately, QUS has the potential to transform MASLD management by offering a cost-effective, scalable, and real-time imaging solution. With continued technological innovation and collaborative validation, it may evolve into a cornerstone of noninvasive liver diagnostics.

As illustrated in **Table 3**, the choice of QUS modality can be aligned with disease stage to optimize diagnostic yield—from fat detection in early MASLD to perfusion and stiffness mapping in advanced fibrosis.

**Table 3:** Stage-based QUS recommendations for MASLD.

Disease stage	Recommended techniques	Clinical role
S0–S1 (Early steatosis)	B-mode ultrasound, ATI, TSI	Initial screening and detection of mild hepatic fat accumulation
S2–S3 (Moderate–severe steatosis)	CAP, TAI, UGAP, UDFF	Quantitative assessment of hepatic fat burden and monitoring of steatosis progression
F1–F3 (Fibrosis progression)	SWE, TE	Quantitative assessment of liver stiffness and risk stratification for advanced fibrosis
Advanced disease with inflammatory or microvascular alterations	SWD, SMI, UMA (exploratory)	Evaluation of viscoelastic and microvascular changes associated with necroinflammation or disease activity

ATI, Attenuation Imaging,TSI, Tissue Scattering Imaging,B-mode,B-mode Ultrasound,CAP, Controlled Attenuation Parameter,SWE,,Shear Wave Elastography (includes both point-SWE and 2D-SWE),UGAP, Ultrasound-Guided Attenuation Parameter,RTEm, Real-Time Tissue Elastography,SMI, Superb Microvascular Imaging,UMA, Ultrasensitive Microvascular Analysis, These techniques are applied according to disease stage, with a focus on detecting subtle fat accumulation in early steatosis (S0–S1), reliably quantifying fat and stiffness in moderate steatosis/early fibrosis (S2/F1), and comprehensively assessing stiffness, perfusion, and elasticity in advanced disease (S3/F2–F3).

Suggested modalities emphasize fat, stiffness, or perfusion assessment depending on steatosis and fibrosis severity. **Table 3** provides a reference overview of commonly used liver imaging techniques across MASLD stages. The choice of multiparametric assessment in clinical practice is primarily determined by the availability of imaging modalities at the diagnostic center, rather than strict stage-based allocation.

The stage-oriented allocation of QUS modalities summarized in **Table 3** reflects their primary physiological targets rather than strict disease-stage boundaries. Attenuation-based techniques such as CAP, TAI, UGAP, and UDFF are mainly used for the quantitative assessment of hepatic steatosis across a wide spectrum of disease severity. In contrast, elastography-based methods including transient elastography (TE) and shear-wave elastography (SWE) provide reliable evaluation of liver stiffness and are therefore more relevant for fibrosis staging and risk stratification. Emerging techniques such as shear-wave dispersion (SWD), superb microvascular imaging (SMI), and ultra-microvascular analysis (UMA) may provide complementary information on necroinflammatory activity and microvascular remodeling. However, their clinical role in routine management of MASLD remains exploratory and requires further validation in larger studies.

Future efforts should prioritize the development of standardized protocols for QUS acquisition, ensuring cross-platform compatibility and reproducibility across devices. Additionally, AI-based analysis of QUS data should be explored to enhance diagnostic precision and overcome the limitations of human operators. The integration of QUS with other diagnostic tools, such as liver elastography and MRI, will enable a more holistic approach to MASLD diagnosis and management. Large-scale multicenter trials are needed to validate these technologies in diverse patient populations and establish universal diagnostic thresholds.

## Conclusion

In conclusion, quantitative ultrasound (QUS) has emerged as a promising non-invasive diagnostic tool for MASLD, enabling comprehensive evaluation of liver steatosis, inflammation, and fibrosis through acoustic attenuation, elasticity, backscatter, and microvascular flow. Recent advancements in multiparametric approaches and AI-enhanced analytics have significantly improved its diagnostic accuracy, offering a robust platform for personalized risk stratification and management of MASLD. However, challenges such as platform standardization, inter-vendor variability, and limited large-scale validation remain. Future research should focus on addressing these challenges, particularly through cross-platform harmonization, the development of universal diagnostic thresholds, and the integration of AI to enhance the clinical applicability of QUS. Ultimately, QUS, with its potential for multi-modal integration, promises to become a cornerstone for the non-invasive monitoring and management of MASLD in clinical practice.

Future research should focus on addressing issues of standardization, cross-platform application, and multiparametric integration to improve the clinical utility of QUS in routine screening and long-term monitoring of MASLD, particularly in settings with limited access to MRI or biopsy.
