# Correction: Advances in quantitative ultrasound for metabolic dysfunction-associated steatotic liver disease diagnosis

**DOI:** 10.3389/fphys.2026.1876129

**Published:** 2026-05-26

**Authors:** Zejun Ma, Bo Liu, Shuyu Zhou, Shuai Yang, Xiaojie Sun, Xinping Jiang, Xiaofeng Sun

**Affiliations:** 1 Department of Cadre’s Wards Ultrasound Diagnostics, Ultrasound Diagnostic Center, The First Hospital of Jilin University, Changchun, China; 2 The First Hospital of Jilin University, Changchun, China

**Keywords:** artificial intelligence, attenuation imaging, liver physiology, MASLD, quantitative ultrasound, shear-wave elastography

Author “Shuyu Zhou” was erroneously spelled “Shuyi Zhou”.

The original version of this article has been updated.

