# Supplementary figures and images for: Advances in quantitative ultrasound for metabolic dysfunction-associated steatotic liver disease diagnosis
Source: Front Physiol. 2026 May 8;17:1802284. doi: 10.3389/fphys.2026.1802284 (PMC13247686; doi:10.3389/fphys.2026.1802284)

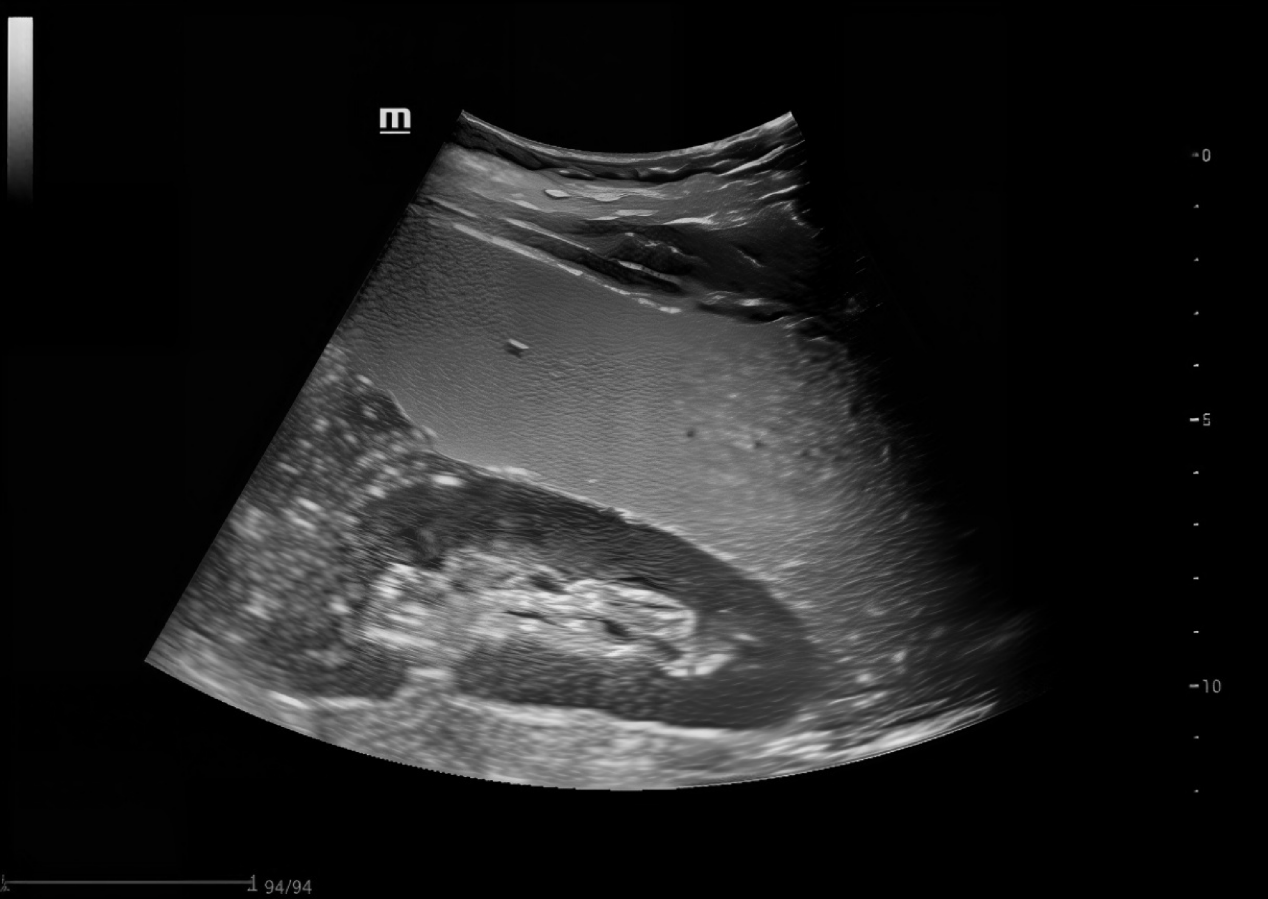

Supplement: Supplementary file 1 [file Image1.png]

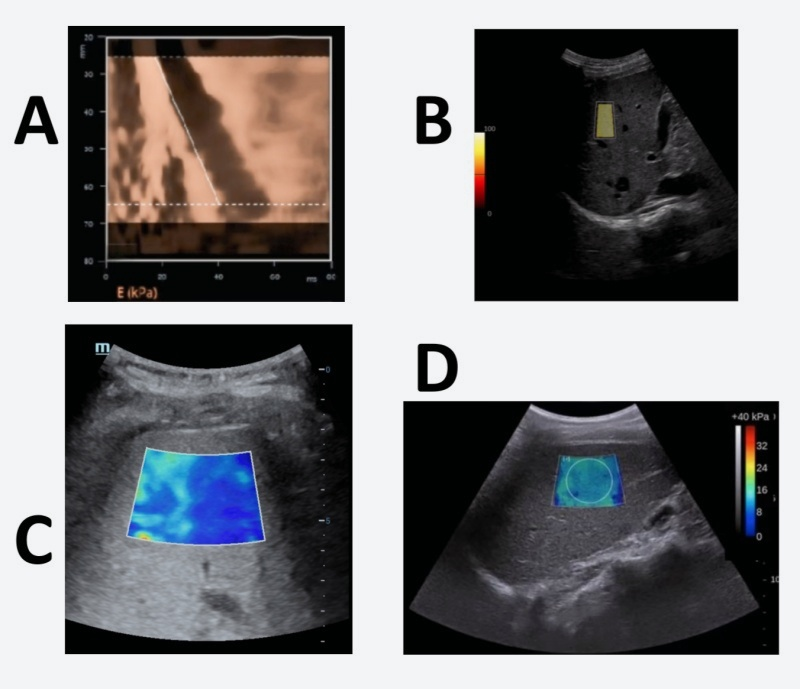

Supplement: Supplementary file 2 [file Image2.png]

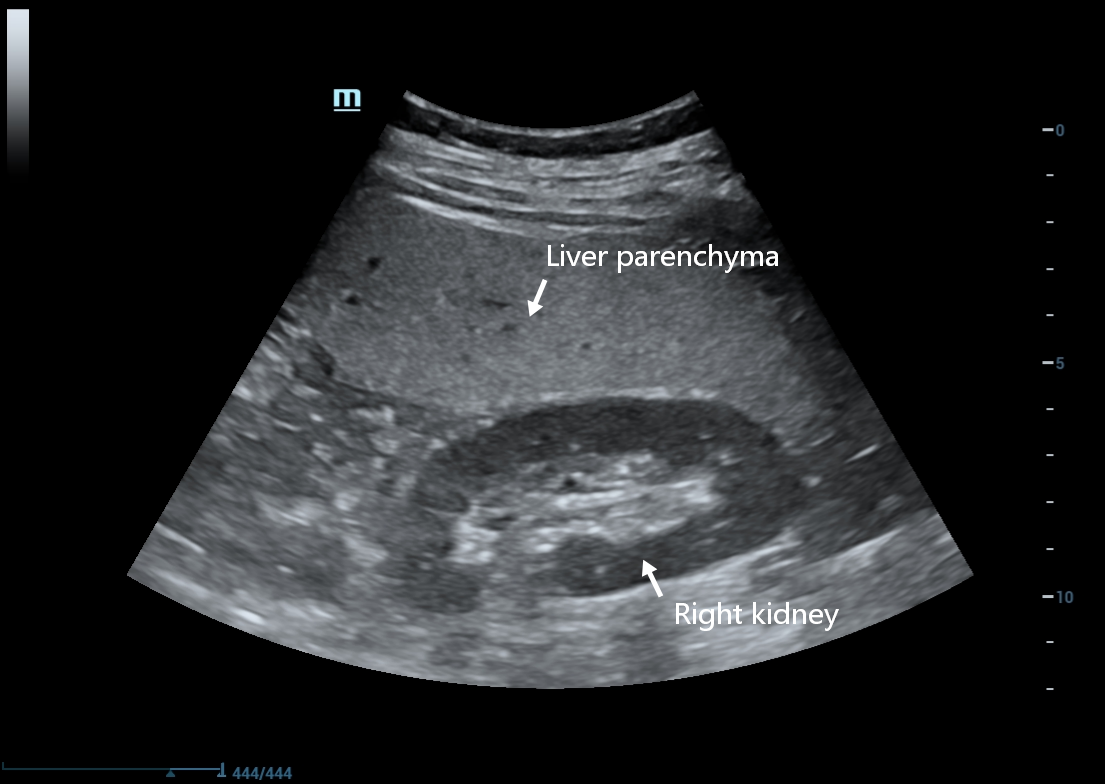

Supplement: Supplementary file 3 [file Image3.png]

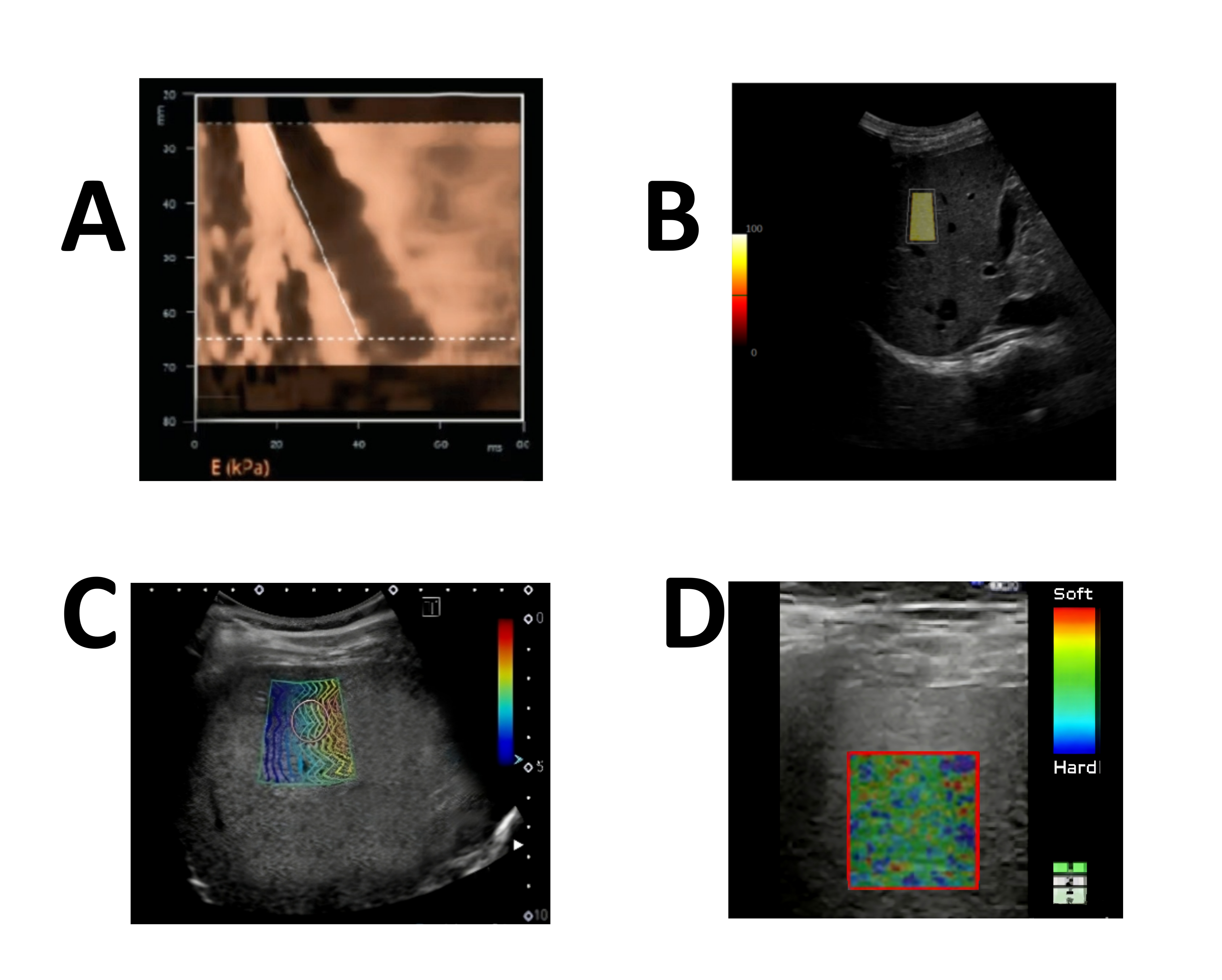

Supplement: Supplementary file 4 [file Image4.png]

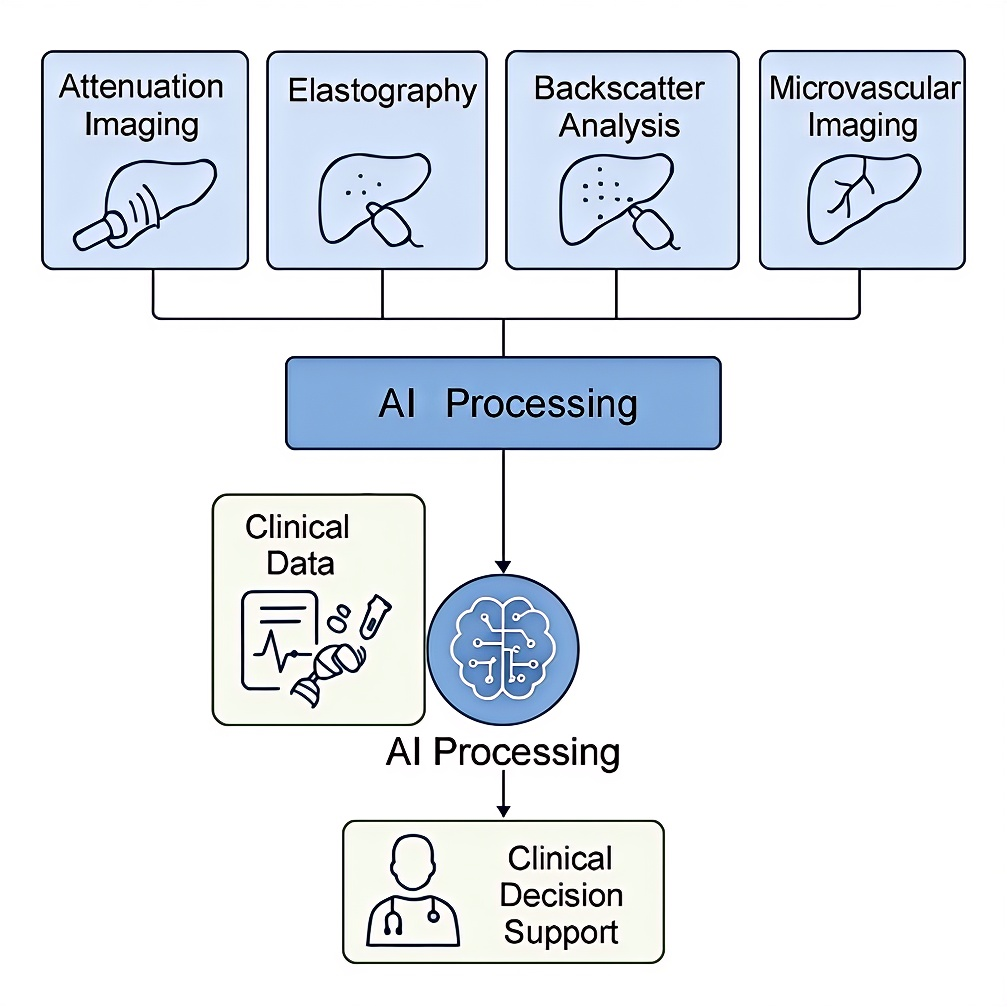

Supplement: Supplementary file 5 [file Image5.png]
